# Ritual responses to drought: An examination of ritual expressions in Classic Maya written sources

**DOI:** 10.1007/s10745-018-0019-6

**Published:** 2018-09-14

**Authors:** Eva Jobbová, Christophe Helmke, Andrew Bevan

**Affiliations:** 1 Institute of Archaeology, University College London, London, UK; 2 Institute of Cross-cultural and Regional Studies, University of Copenhagen, Copenhagen, Denmark

**Keywords:** Epigraphy, Agriculture, Precipitation, Ritual, Maya, Belize, Guatemala, Eastern Mexico

## Abstract

Planting and rain-beckoning rituals are an extremely common way in which past and present human communities have confronted the risk of drought across a range of environments worldwide. In tropical environments, such ceremonies are particularly salient despite widespread assumptions that water supplies are unproblematic in such regions. We demonstrate for the first time that two common but previously under-appreciated Maya rituals are likely planting and rain-beckoning rituals preferentially performed at certain times of the year in close step with the rainy season and the Maya agricultural cycle. We also argue for considerable historical continuity between these Classic Maya ceremonies and later Maya community rituals still performed in times of uncertain weather conditions up to the present day across Guatemala, Belize, and eastern Mexico. During the Terminal Classic period (AD 800-900), the changing role played by ancient Maya drought-related rituals fits into a wider rhetorical shift observed in Maya texts away from the more characteristic focus on royal births, enthronements, marriages, and wars towards greater emphasis on the correct perpetuation of key ceremonies, and we argue that such changes are consistent with palaeoclimatic evidence for a period of diminished precipitation and recurrent drought.

## Introduction

The Maya are one of the best-known civilisations of Mesoamerica, noted for their art, architecture, astronomy, mathematics, calendrical systems, and their hieroglyphic script – one of the few fully developed writing systems of the pre-Columbian Americas. Maya Classic period (AD 250-900) texts are well-known for their commemoration of the passing of time and are focused especially on the deeds of kings, including royal births, enthronements, marriages, rituals, and wars. However, Terminal Classic (AD 800-900) texts are something of an exception in remaining essentially mute about the warfare and social upheavals that other archaeological evidence suggests were pronounced at this time. Instead, Terminal Classic texts constantly emphasise ritual continuity via the proper perpetuation of key ceremonies. This narrative and rhetorical shift in the last century or so of the Classic period is not only interesting in its own right, but also implies a growing disjunction between what was actually taking place and what the texts relate. Given this dissonance, it is worth asking why this narrative change appears at precisely this time in Maya history, what was the nature of the rituals the texts record, and what these ceremonies tell us, directly or obliquely, about the preoccupations of the Terminal Classic Maya?

Although the relationship between records of royal ceremonial performance and the wider ecological and agricultural concerns of Maya society has been discussed before (e.g., Freidel and Shaw 2000; Lucero 2006; Schaafsma and Taube 2006; Dunning and Houston 2011), in this paper we explore the relationship in a novel way by combining multiple sources of evidence (epigraphic, ethnographic, palaeoclimatic, and modern rainfall data) in order to examine possible links between specific Maya rituals and periods of environmental stress. The increasing range of palaeoclimatic archives indicating diminished precipitation and even recurrent severe droughts during the Terminal Classic provides context for our discussion (Brenner *et al*. 2002; Leyden 2002; Rosenmeier *et al.* 2002; Webster *et al*. 2007; Wanner *et al.* 2008; Kennett *et al*. 2012, Douglas *et al*. 2015). Such evidence undermines the perception of people from temperate climates that the humid tropics are characterized by abundance of water. To people who live there, variable rainfall patterns–too little or too much rainfall per rainy season, enough rainfall but at the wrong time, or a series of long dry seasons–have always been critical issues.

**Fig. 1:**
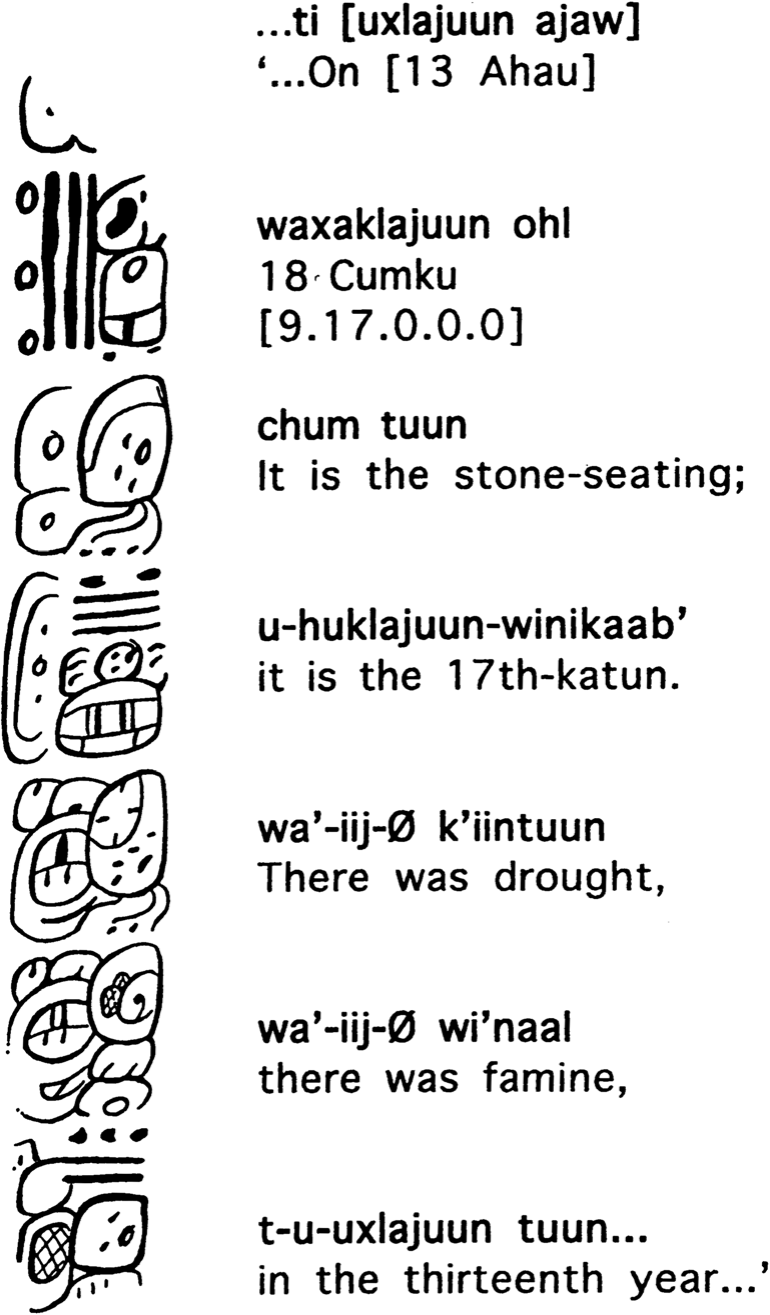
Inscribed Stingray Spine 3, of Urn 26 from Comalcalco, which records an event of drought and famine in AD 783 (after Zender 2004: 543, Fig. 73)

We also provide context and greater time depth for the attention paid to food production crises in much later Maya literature, such as prophecies recorded in the *Chilam Balam* books (dated mostly to the seventeenth and eighteenth centuries AD; Roys 1967: 122; Edmonson 1986; Bricker and Miram 2002) or the Dresden and Paris Codices (dated to the twelfth and thirteenth centuries AD; Love 1994; Grube 2012). Whereas earlier Maya hieroglyphic inscriptions focus mainly on royal life, texts referring directly to drought do exist, although perhaps surprisingly there are only two such references in the thousands of known Classic period texts. The first is a hieroglyphic text from the site of Comalcalco (in present-day Tabasco, Mexico), from the final resting place of a Maya priest named *Ajpakal Tahn*, whose burial urn was richly furnished with jade jewellery, shark’s teeth, carved shell and human bone pendants, obsidian blades, a flint eccentric, and stingray spines with glyphic texts as well as iconographic scenes (Armijo Torres 1999; Armijo Torres and Zender 1999; Armijo *et al.* 2000; Zender 2004: 250). One of the stingray spines bears a text that says rather uncompromisingly: ‘there was drought, there was famine in the thirteenth year’ (Zender 2004: 257, 543), which, based on associated calendrical notations, places the drought in the latter half of the eighth century^1^ (Fig. 1). The second is a prophetic rather than historical reference that is found on the Central Tablet of the Temple of Inscriptions at Palenque, which may refer to a drying out or to the ‘withering’ of the World Tree (Lacadena 2006; Guenter 2007: 32). These two texts confirm a deeper history of drought in the region, but raise the question of whether these two references to drought and famine are all that it is to be found in the hieroglyphic record, or whether we are overlooking some indirect evidence?

Conspicuous investment in ritual practice is a widespread human response to periods of climatic stress, with rain-beckoning during episodes of drought being especially common cross-culturally. While also considering the wider character of the Classic Maya glyphic corpus, we place considerable emphasis on two particular rituals, one involving the ‘scattering’ of precious substances and the other a ‘bathing’ ritual involving a particular pair of deities. These are in fact the two most commonly-recorded rites in the Terminal Classic period, and we argue below that they were both closely related to the yearly agrarian cycle, respectively symbolizing the act of sowing and the invocation of rain-bearing clouds. Beyond this general agricultural and ecological link, we examine the temporal and spatial distribution of these rituals, which suggest a close association with periodic as well as protracted droughts.

## Rain-beckoning rituals

Ritual activity as a mechanism for dealing with environmental stress has been discussed in many ethnographic and archaeological studies worldwide (e.g., Frazer 1911; Butree 1930), but also specifically in the Maya area. For instance, Nash (1970: 45) discussed cave rituals to beckon rain performed in Chiapas during the times of drought. Similar rituals have been reported also among the Tzotzil of Zinacantan (Gossen 1999: 185) as well as Nahua and Otomi people in Veracruz (Sandstrom 2005), and the use of ritual as a coping strategy to anticipate and mediate risk among the Lowland Maya has been discussed by Freidel and Shaw (2000). Various ritual obligations related to agriculture in the Maya area are also documented (e.g., Wilk 1991; Tzul 1993; Flores and Balam 1997; Hatse and De Ceuster 2001; Grandia 2004). Recent ethnographic fieldwork at six different villages in the Cayo and Toledo districts of Belize (Downey and Jobbová 2011) gathered first-hand information from local informants about recent historical climate variability, experiences of drought, and short-term responses to changing weather patterns^2^ and provides important modern context for such Classic Maya practices. A further goal of this research was to identify modern-day ceremonies related to drought or other types of climate stress, to establish their time-depth and determine whether such practices persisted in Belize into the twentieth century. The results indicated a variety of coping strategies with regard to environmental disaster or stress (especially drought, but also locusts, and hurricanes), including a surprising variety of rituals that could be enacted during periods of drought. While this ethnographic study found many local differences among accounts of general-purpose and drought-related rituals across the two study regions of Belize, one or two documented modern rituals exhibited greater coherence, of which perhaps the most interesting is a rain-beckoning ceremony called *Ch’a-cháak* known especially in the Yucatan, but also in parts of northern and central Belize. Clearly, this ritual is related to *Chaahk*, the ancient Maya deity of rain, the personification of thunder and associated with rain and clouds (see Stone and Zender 2011: 41; Wrem Anderson and Helmke 2013). One ritual, however, was described in nearly identical ways in both study regions: if it did not rain, the village’s saint was taken out from the church and ‘bathed’ in a spring, or left out in the sun until the stone started to sweat at which point the water was poured over it. Interestingly, the eminent Mayanist Sir J. Eric S. Thompson documented a similar ritual during his ethnological research in the 1930s at San José Succotz (Cayo district) and San Antonio (Toledo district). In describing the rain-beckoning ceremonies he writes:

If the prayers for rain are not effective, the Mayas call the attention of the saints to the drought. Any saint from the church is taken outside and placed well in the sun, so that he or she may be convinced how parching are the hot rays of the sun. Undoubtedly in earlier times a statue of one of the rain gods was the victim of this irreverent treatment. At present no statues of the old gods survive and the Christian saints have to suffer in their place. Sometimes the saint is taken out of the church and marched around the building, while prayers are offered to Huitz-Hok and Santa U [the moon]. The previous night is passed in vigil (1930: 53).

The use of effigies in agricultural and/or specifically rainmaking rituals has a long tradition and is geographically widespread. In ancient Egypt, Mesopotamia, Sumer, Hittite Anatolia, Classical Greece and Rome, such ceremonies involved god effigies being carried to a river and washed, often followed by a sacrificial offering and a communal meal (Başgöz 1967: 305). Many aspects of these rituals are still used in rainmaking-ceremonies today, or have persisted until recently. For example, in Turkey children make a doll, carry it around the village, and at each house water is poured over the head of the doll. They are offered food, which is then cooked and eaten (Başgöz 1967: 304).

**Fig. 2:**
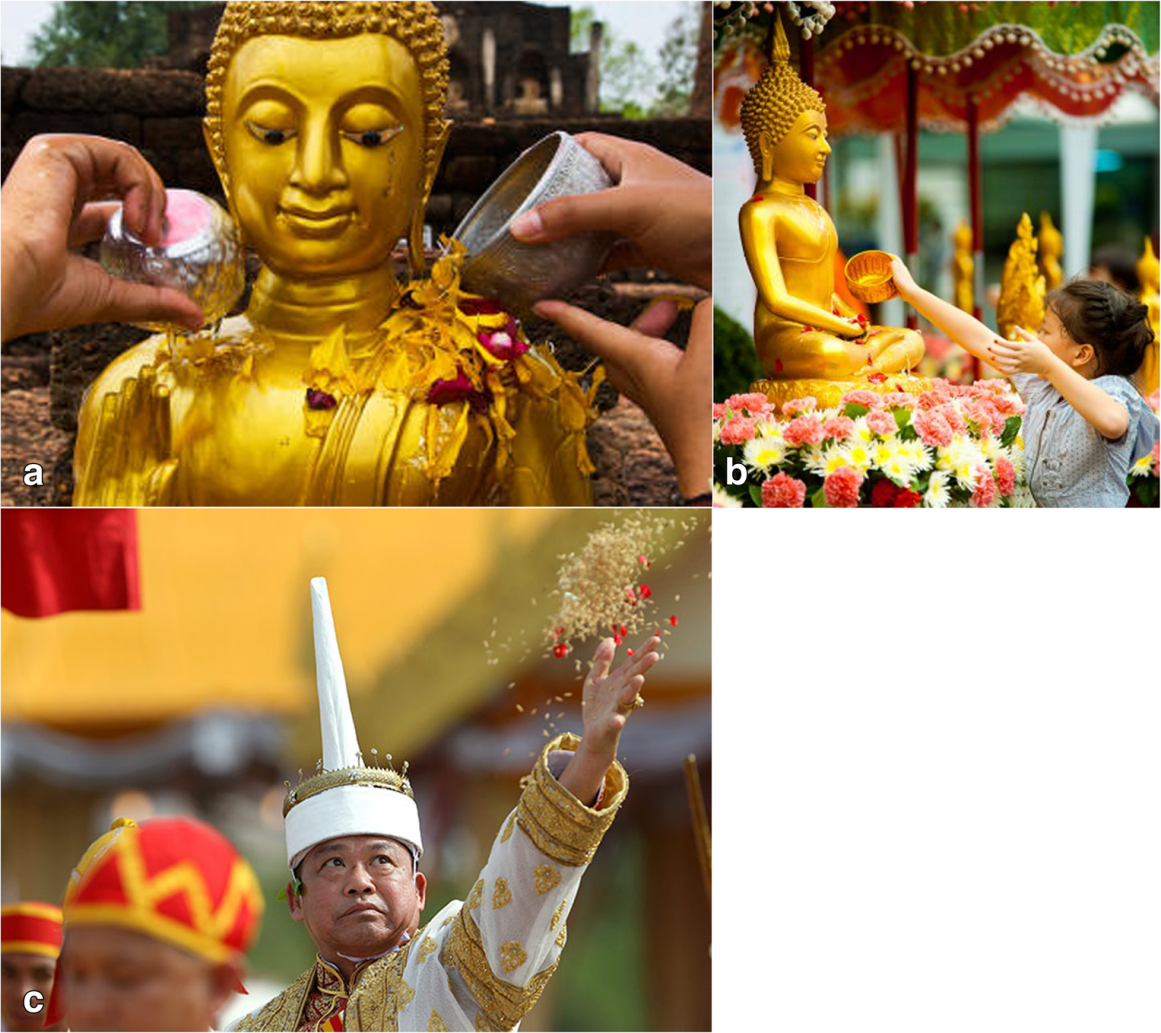
Bathing and sowing rituals in South-East Asia. **a**) Bathing a statue of the Buddha in Laos with scented water. **b**) The comparable *Songkran* ritual in Laos. **c**) The royal ploughing ceremony in Thailand (images in the public domain)

Emphasis on rainmaking rituals is also obvious in past and present activity in other tropical environments such as south-east Asia. One widely celebrated Southeast Asian holiday is the *Songkran* festival (from the Sanskrit word *saṃkrānti* describing astrological passage, and marking the beginning of a new Solar year; Monier-Williams 1899: 1127). This New Year festival, under different names, is celebrated, for example, in Burma, Cambodia, and Laos (Fig. 2a), but can be traced back to India and Hindu rituals. It is performed in the middle of April (Fig. 2b), corresponding to the hot and dry period of the year, before the start of the monsoon, when people are praying for good rainfall and abundant harvest in the upcoming season. During the *Songkran* festival people cleanse Buddha images by pouring scented water over them and smaller effigies are often taken out of the monasteries and carried through the streets while people sprinkle water at them (Milne 1924; Rajadhon 1956, 1958, 1961; Ashley 2005). Similar rituals, with slight variations, are performed during the *T’ngai Leang Saka* (the third day of a Cambodian New Year) and *Boun Pi Mai* (Laotian New Year) (Rajadhon 1961; Chiebriekao 2008).

In Thailand and Cambodia, a royal ploughing ceremony is called *Phra Rat Cha Phithi Charot Phra Nangkhan Raek Na Khwan* (literally the ‘royal ploughing ceremony marking the auspicious beginning of the rice growing season;’ Royal Institute Dictionary 1999) (Fig. 2c). It is an ancient ritual of Hindu origin, dating back to the Sukhothai period (AD 1238-1438) and usually taking place in May, June, or July, the exact date being set by Brahmin astrologers of the royal household. During this ceremony the king, as lord of the harvest, tills the ground with a plough pulled by sacred bulls. At the end of the ceremony, the king scatters rice over the ploughed furrows, which is then quickly gathered by people who believe that it will ensure a good harvest. More than a religious ritual, the ploughing ceremony is a state event that has both political and economic significance, functioning as a reminder of a bond between the king and farmers (Crawfurd 1830). This same ceremony has also been recorded in Burma as one of the rain invoking rites, which the king himself is obliged to perform in order to prove his nobility and illustriousness, with such royal actions emphasising his role as a ‘Peasant King’, ‘one of them,’ and theoretically inspiring peasants to work hard for a plentiful harvest (Maung Nyunt 1997). It is worth noting the differences between Songkran rain festivities and the southeast Asian royal scattering/ploughing ceremony in terms of the greater agency given to ordinary people in the first case, but the more hierarchical, royal interventions involved in the second. One reason may be the interest the king and state might have in rice as a taxable commodity, much as Maya kings may have had with respect to maize.

## Possible Classic Maya parallels

The ceremony described by Thompson (1930) above provides a link between contemporary rain calling rituals and those he recorded in the 1920s, but it is very likely that at least some of these rituals or certain aspects of them might have survived from pre-Columbian times. Several anthropologists and archaeologists focusing on contemporary Maya rituals have commented on the extent that pre-Columbian beliefs can still be detected (e.g., Thompson 1930; Vogt 1976, 1998; Schuster 1997). Ethnologist Evon Vogt said that ‘considering that 500 years have elapsed since the Spanish Conquest, I am impressed with the enduring nature of Classic Maya concepts and beliefs’ (cited in Schuster 1997: 50). Furthermore, ritual theorist Pierre Smith (1982) made a distinction between ‘periodic’ and ‘occasional’ rites. Whereas periodic rituals are performed cyclically, occasional rituals are performed on an ad hoc basis, thereby increasing ritual frequency. For example, increased frequency of ritual activity in times of environmental stress among the Maya has been documented by several ethnographic studies (e.g., Girard 1949, 1995; Freidel and Shaw 2000). More important for this particular study are recent studies of material remains in caves of Western Belize (Moyes 2006; Helmke 2009; Helmke *et al*. in press), which have shown that there is an evidence of increased ritual activity in caves during the latter part of the Late Classic period (ca AD 680-960) coincident with climatic drying. Based on the evidence, authors of these studies argue that this ritual activity can be associated with rain-making and agricultural security. This emphasis on ritual activity in the archaeological record corresponds well with increased ritual focus of Terminal Classic Maya texts.

**Fig. 3:**
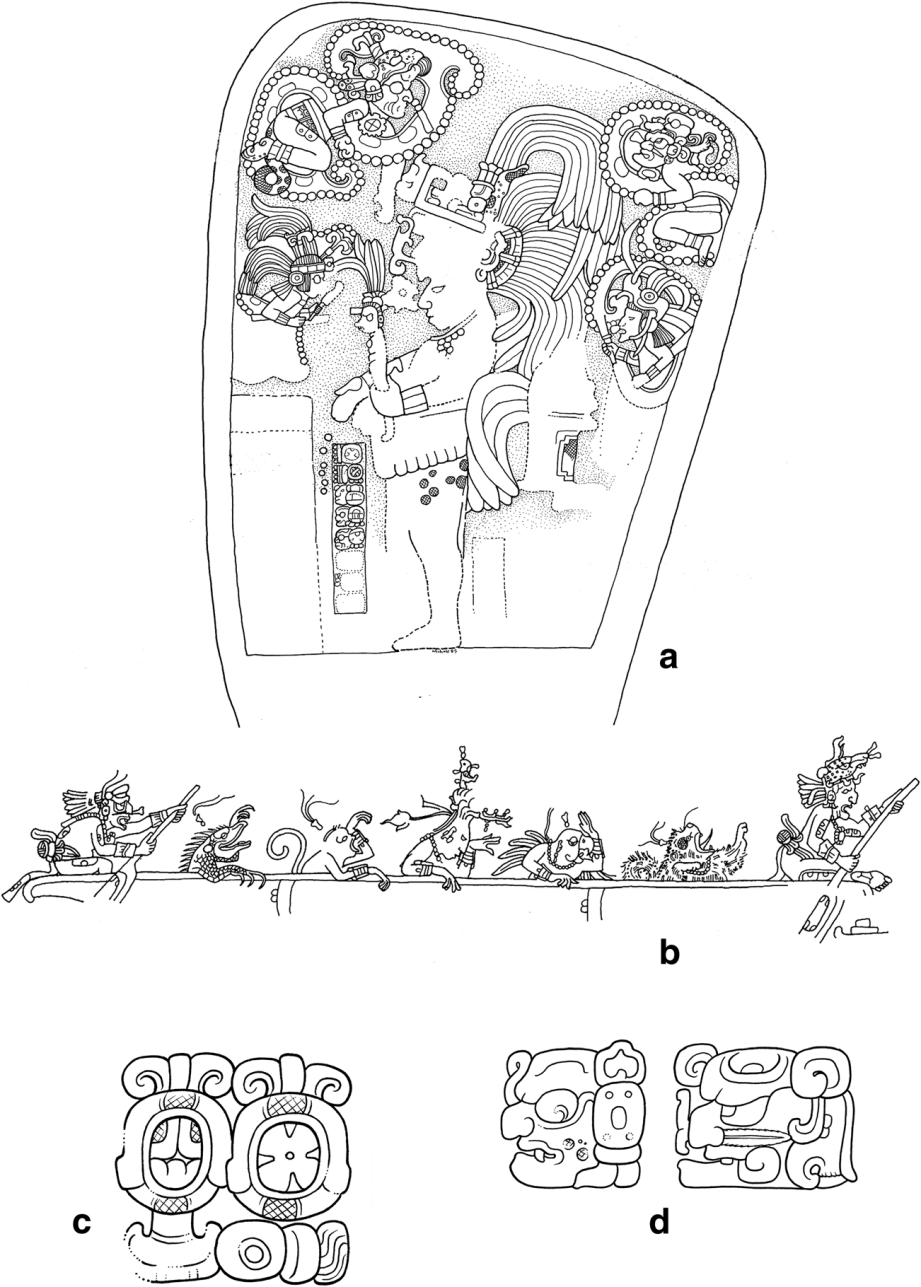
The Paddler deities in ancient Maya imagery and writing. **a**) Stela 2 at the site of Ixlu dated to AD 879 in the Terminal Classic. **b**) The Paddler deities ferrying the deceased Maize god to the watery underworld (drawings by Linda Schele © Los Angeles County Museum of Art). **c**) The name glyphs of the Paddler deities in the shape of diminutive and stylized paddles (Stucco text at Tonina). **d**) The name glyphs of the Paddlers representing their profiles and characteristic traits (Stela C at Quirigua) (drawings by Christophe Helmke)

Among ceremonies commemorated on Classic Maya monuments, there are two that prevail during the Terminal Classic and that we examine in detail below. The first involves the ‘scattering’ of precious substances and the other the ‘bathing’ of a particular pair of deities, known as the “Paddler Gods” (Schele and Miller 1986: 52, 183; Freidel *et al.* 1993: 91-94; Stone and Zender 2011: 51, 69; Stuart 2016). A good place to start is with the stelae from the sites of Ixlu and Jimbal where we can see depictions of the Paddler deities in the upper portions of the scene, amidst dotted-scroll motifs that represent clouds, floating above the king, who performs a ‘scattering’ ceremony (Schele and Miller 1986: 52, 183; Stuart *et al.* 1999: 169-70) (Fig. 3a). The Paddlers are an important pair of Maya deities whose names remain undecipherable. One is nicknamed the Old Jaguar paddler, recognizable by his jaguar spots and the ear of a feline, whereas the other, the Stingray Paddler has a prominent stingray spine or sharpened bone piercing his septum (Fig. 3d). Their names are often represented in the glyphs as signs that resemble diminutive and stylised paddles, wherein the one is qualified by a sign for *k’in* (‘sun, day,’ perhaps ‘light’) and the other by *ak’bal* (‘night’) or *ahk’ab* (‘darkness’) (Stuart 1984: 13-15) (Fig. 3c). These deities are often depicted paddling a long dugout canoe, as for example in the scene incised on a human bone found in Burial 116 at Tikal, Guatemala, where the Paddlers ferry the deceased Maize God across the waters of the underworld to a place of resurrection (Freidel *et al*. 1993: 92; Stone and Zender 2011: 51) (Fig. 3b). From other texts, we know that this pair of Maya deities is associated not only with the creation of the world (Freidel *et al*. 1993: 92) but also with rain. Stela 1 from the site of Jimbal, for instance, specifically mentions *Chaahk*, the Maya deity of thunder and rain in connection with the names of the Paddler Gods.

In the ritual depicted on Stela 2 of Ixlu, the Paddler Gods are depicted floating within dotted-scrolls (Fig. 3a). Earlier studies identified this scroll motif as representing blood (Schele and Miller 1986: 52, 183; Stuart 1988: 184) and thus the Paddlers were thought to be born from the blood of the king’s auto-sacrifice (Stuart 1984: 14-15; Schele and Miller 1986: 52, 183). Nevertheless, with the decipherment of the glyph for *muyal* ‘cloud’ in Classic Maya, the dotted-scroll motif is now understood as representing clouds, both of rain and incense, the two being symbolically equivalent (Houston and Stuart 1990). More recently, Stuart and Houston have suggested that these scenes depict the Paddler Gods undergoing a ‘bathing’ ritual, as a kind of rite of purification, possibly related to ‘rainmaking rituals’ (Stuart *et al*. 1999: 169-171).

**Fig. 4:**
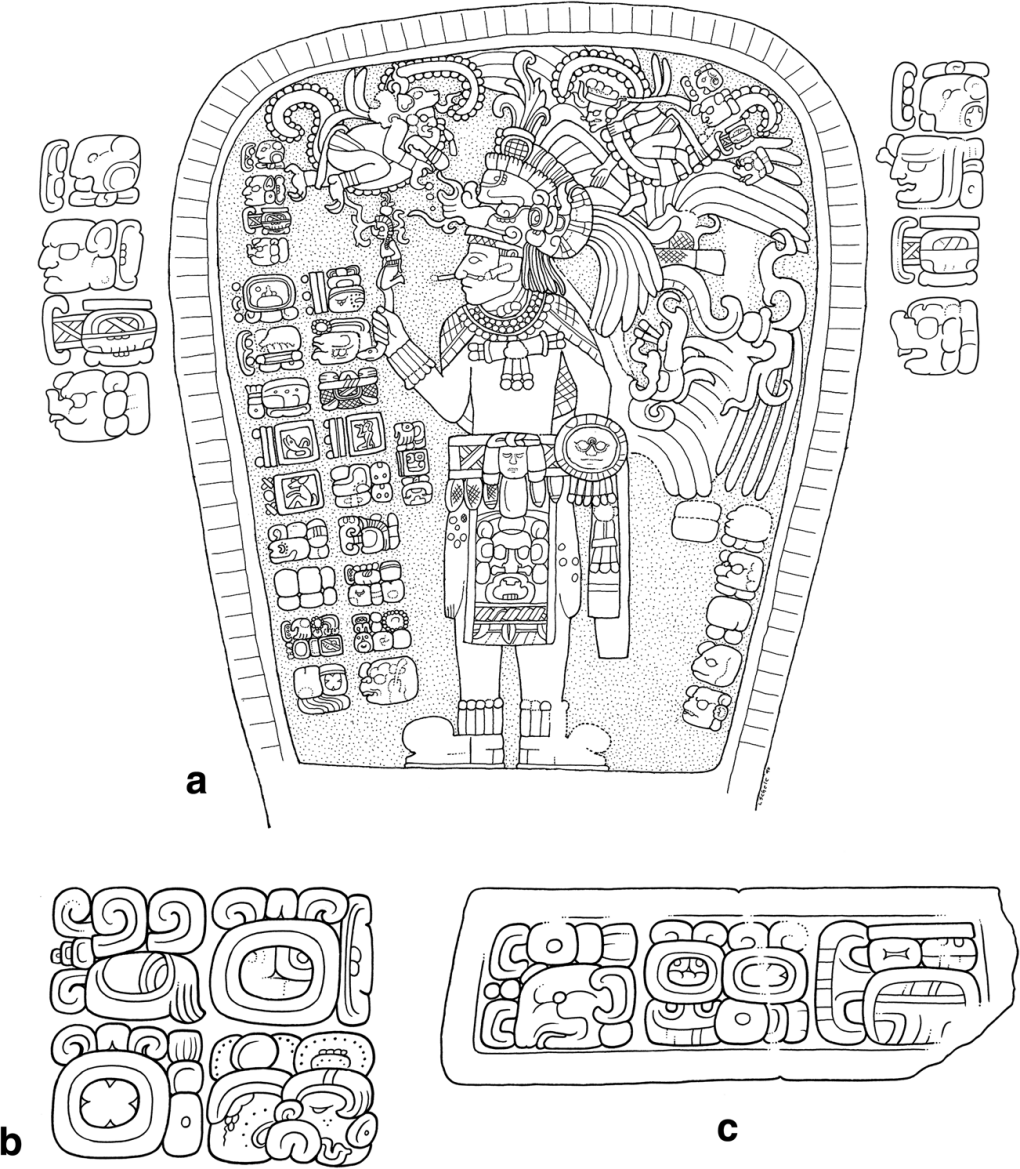
**a**) Jimbal Stela 1, dated to AD 879 in the Terminal Classic. Note the paddler deities floating above the king who brandishes his sceptre. The glyphic captions name each of the Paddler deities as a *Chaahk* entity (final glyph in each caption), personifying rain and thunder (drawing Linda Schele © Los Angeles County Museum of Art; glyphic captions drawn by Christophe Helmke, based in part on photographs of the Atlas Epigráfico de Peten, courtesy of Dirección de Patrimonio Cultural y Natural de Ministerio de Cultura y Deportes, Guatemala). Examples of the *yatij* ‘bathing’ expression connected to the Paddler deities in Classic Maya writing. **b**) Side of Stela 22 at Naranjo (**ya-AT-ji**) where the Paddler deities are qualified as *junpik k’uh* ‘eight thousand gods’. **c**) Detail of Monument 42 at Tonina (**ya-ti-ji**) that provides the Paddlers with the title *Nahho’ Chan Ajaw* or ‘kings of the First Five Skies’ (drawings by Christophe Helmke, after drawings by Ian Graham and Peter Mathews)

Examination of the texts that accompany these scenes led Stuart and Houston to note the close affinity between the Paddler Gods and a particular verbal statement (Fig. 4b-c) that is usually written as **ya-AT-ji** or **ya-ti-ji** (the latter phonetic spelling has enabled the decipherment of the more common logographic spelling, Stuart *et al*. 1999: 169; David Stuart, pers. comm. 2000). Analysed as *y-at-ij,* the root of this verbal expression is *at* ‘to bathe,’ which is interpreted as a nominalised construction (marked by the suffix) with a possessive prefix. This would prompt the translation of ‘it is the bathing of the Paddler Gods’ (Prager 2013: 261), in the passive mood, assuming that *at* is intransitive, based on Ch’olan cognates (Stuart *et al*. 1999: 169). Nevertheless, the constructions involving this expression and the Paddlers may also be interpreted as transitive constructions, involving a perfective suffix –V*j*, not least since the cognate *atih* in Ch’orti’ is the transitive form of ‘bathe, wash’ (Wisdom 1950: 454). On this basis, the clause would be translated as ‘the Paddler Gods bathe(d)’ (MacLeod 2004: 294; Alfonso Lacadena, pers. comm. 2013).

**Fig. 5:**
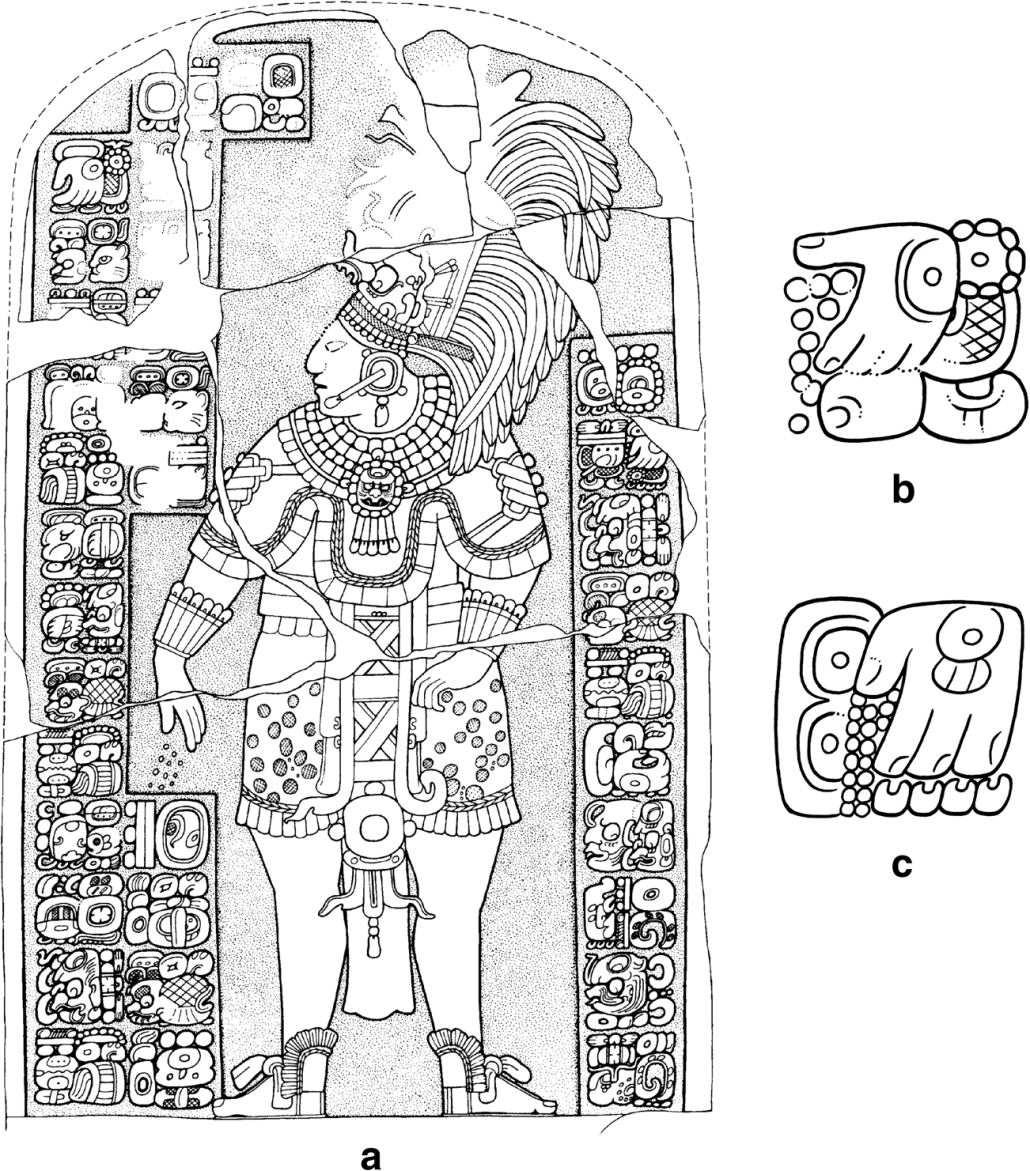
The *choko’w ch’aaj* ‘the scattering of drops’ ritual in Classic Maya imagery and writing. **a**) Stela 1 at Aguateca that shows the local king performing a scattering ritual in AD 741 (drawing by Ian Graham, after Graham 1968: Fig. 3). **b**) The verbal root of this ritual is represented in writing by a hand that scatters drops (written **CHOK-wa-ch’a**) on Arroyo de Piedra Stela 2. **c**) The same expression (written **u-CHOK-ji**) on Tonina Mon. 104 (drawings by Christophe Helmke)

The second, ‘scattering’ ritual is more clearly part of a longer tradition, with a relatively wide geographical distribution across Mesoamerica. It is often represented both in iconographic and glyphic form. In iconographic form, it appears probably as early as 900 BC on the Humboldt Celt, where Justeson (1986: 443) interpreted it as the ceremonial casting of maize kernels. The scene usually involves a ruler with outstretched arms and open hands throwing or scattering small round objects (Fig. 5a). In written form, this action is represented by a glyph depicting an open hand with small dots falling from it (Fig. 5b-c). The early glyphic form may be recorded in Isthmian writing on La Mojarra Stela 1 in Veracruz dating to second century AD (Justeson and Kaufman 1993: Fig. 6). Many Early Classic examples are also known from Teotihuacan, the great metropolis in the central Mexican highlands (e.g., Helmke and Nielsen 2014: 89-91, 93, Figs 9a-b, 11). In Maya writing the glyph in question is read *chok*, meaning ‘to scatter, sprinkle’ (Stuart 1984: 9; Schele and Grube 1995: 40). While this reading is clear, there remains on-going debate about what the bead-like objects falling from the hand represent, with suggestions that they are droplets of water (Kelley 1962: 40; Dütting 1974: 50; Thompson 1962: 300f), grain (Thompson 1962: 300f; Justeson 1986: 443; Proskuriakoff 1993), blood (Stuart 1984: 9, 1988: 187-8; Schele and Miller 1986: 181-182), incense pellets (Love 1987: 11-14) or a combination of these (see Landa in Tozzer 1941: 140-144).

**Fig. 6:**
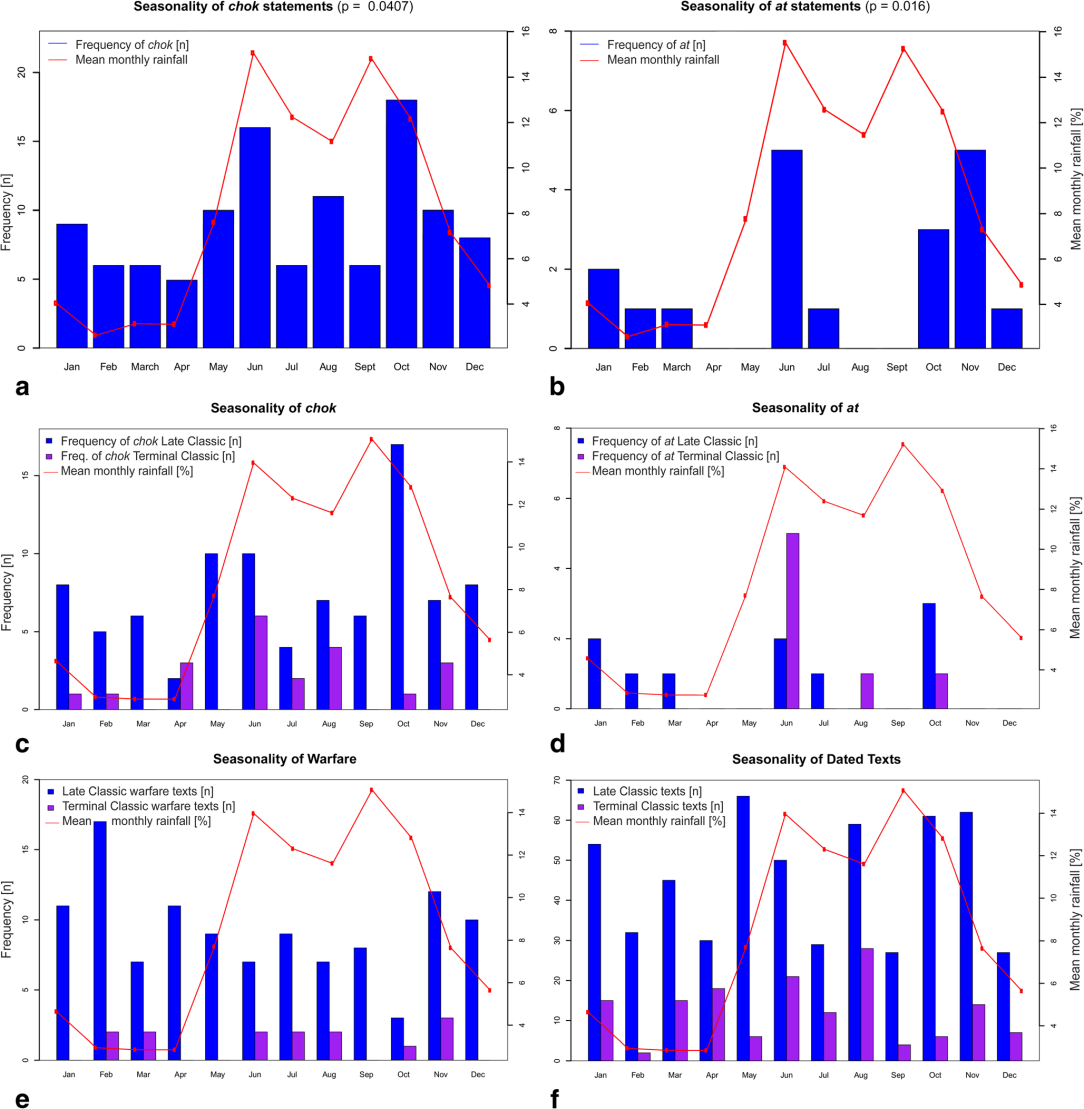
Comparison between the frequency and seasonality of rituals and other types of events against mean monthly rainfall. The mean rainfall values are calculated from weather stations in regions with *chok* and *at* statements for approximately 1950-2000 (www.worldclim.org) and are used as a proxy for the distribution of rainfall throughout the year (for distribution of rainfall in Maya lowlands see Fig. 11b). **a**) Seasonality of *chok* statements. **b**) Seasonality of *at* statements. **c**) *chok* and **d**) *at* statements in comparison to seasonality of **e**) warfare statements and **f**) the incidence of known dated texts, contrasting the Late Classic incidences against those of the Terminal Classic

On the basis of these suggestions and juxtaposing the ‘scattering’ and the ‘bathing’ rituals with the observations from the cited ethnohistorical studies, we propose the following hypotheses:

1. As acts symbolising the sowing of crop seed, and the invocation of rain-bearing clouds, these rituals were closely related to the agrarian cycle. As such, the ‘bathing’ rituals may be the precursors, or proto-forms, of some of the later rain-beckoning ceremonies described above, such as the ‘bathing’ of the village’s saint, or the ceremonies known among the Yukatek Maya as *ch’a-cháak*, attested in both the ethnohistoric and ethnographic sources (i.e., Rejón García 1905; Gann 1917; Irigoyen 1976; Love 1984, 2011; Freidel *et al*. 1993). They are rain-beckoning rituals and at present are performed annually at the end of the dry season, immediately preceding planting and sowing. Similarly, the ‘scattering’ rituals might be also interpreted as related to agricultural ceremonies, celebrating in their emulation, the cycles of planting and sowing of grains on the open field.
2. These rituals may have originated not just in response to general fears about water scarcity, but also to particular episodes of drought and, without pre-supposing this conclusion, it should be considered whether they might not therefore provide historical markers of time periods with diminished precipitation.

## Analytical Approach and Data

In order to test the above hypotheses, we have reviewed the entire corpus of hieroglyphic texts, noting all known occurrences of these two particular kinds of rituals: ‘the scattering of drops’ or *uchoko’w ch’aaj* in Classic Maya and ‘bathing’ or *yatij.* As there is some diversity in the manner in which these two expressions are recorded in the glyphic texts we refer to each in the remainder of this text by the verbal root of the action: *chok* and *at* respectively. The focus on these two rituals is also advantageous since *at* is best known for the Terminal Classic, whereas *chok* is documented for the entirety of the Classic period but also with a high relative incidence in the Terminal Classic. Thus, rather than focusing on a single ritual action, we are able to compare and contrast the spatial and temporal incidence of these two distinct but symbolically-related rituals, seemingly relevant to the semantics of agrarian practices. Our working database consists of information about the site from which the *chok* and *at* statement originates, the date of the statement (or the particular historical iterance) and also the latest date of the text (to assess the degree of overlap between the written source and the event recorded, or whether the historical iterance is highly retrospective). Altogether, 23 *at* (‘bathing’) statements can be recognized from inscribed monuments at seven different sites across southern Mexico, Guatemala, and western Honduras. Of these, 19 can be securely dated (Table 1). We also have added four further monuments that are missing explicit written references to ‘bathing’ but which have scenes clearly depicting this ritual iconographically (e.g., Ixlu, Stela 2; Jimbal, Stela 1) (Table 2). For *chok* (scattering) rituals, there are as many as 124 statements from 38 different sites across southern Mexico, Guatemala, Belize, and western Honduras, of which 112 can be securely dated (Table 3). Below, we first explore the monthly distribution of *chok* and *at* statements, in order to see if there was a preference for performing these rituals during certain seasons (especially given that the Maya solar calendar did not account for annual drift, and as such the emic temporal intervals are not inherently locked to a given seasonality). Ideally, we would explore the seasonality of only those statements with non-period ending dates, which are more likely to represent explicitly special events such as droughts. Owing to small sample size and the dating of the majority of examples to the Terminal Classic, where texts were preferentially raised on period ending dates, this is not feasible. To compensate for this uncertainty, we compare the seasonality of *at* and *chok* statements with the seasonal spread of a much larger set of dated Maya texts undifferentiated by topic (using the database compiled by Guenter [2014], which contains the vast majority of Late Classic and Terminal Classic texts), as well as with other types of events mentioned in texts such as statements about warfare (using Maya Hieroglyphic database ([MHD]; compiled by Macri and Looper [1991] cited in Kennett *et al*. 2012, SM, Table S7) or royal accessions (using a database compiled by Martin 2014). To further elucidate the timing of *chok* and *at* occurrences, we compare these to the seasonal distribution of total rainfall in Maya area. We also compare the longer-term temporal distribution of *chok* and *at* statements throughout the Classic period with palaeoclimatological evidence to explore whether any relationship between the occurrence of these statements and drier periods can be substantiated. Again, to control for possible biases in the overall textual sample, the temporal distribution of *chok* and *at* statements is assessed against the temporal distribution of known dated Maya texts from the vantage of the entire glyphic corpus (using a database compiled by Prager 2008). In terms of temporal distribution we look at statement distribution both throughout the time of their existence (Early Classic to Post-classic) to explore their diachronic change, and also separately for Early/Late Classic and Terminal Classic to explore the ways in which their occurrences differ between these two periods.

**Table 1:** Monuments with written statements of “bathing” ritual

Site name	Monument number	Clause date	Julian date	Season	Date C	Text date (dedication)	Gregorian date	Date M	Transcription
Tikal	Stela 40	9.1.13.0.0 6 Ajaw 8 Sotz’	17 June 468	June	468	9.1.13.0.0 6 Ajaw 8 Sotz’	18 June 468	468	**ya-AT-ji?**..Paddlers
Copan	Stela 2	9.10.15.13 0 6 Ajaw 8 Mol	22 July 648	July	648	9.11.0.0.0 6 Ajaw 8 Mol	14 October 652	652	**AT-ti-ji**
Copan	Stela 12	9.11.0.0.0 12 Ajaw 8 Keh	9 October 652	October	652	9.11.0.0.0 12 Ajaw 8 Keh	14 October 652	652	**ya-ti-ji** Paddlers
Copan	Altar Stela 1	9.12.0.0.0 10 Ajaw 8 Yaxkin	26 June 672	June	672	9.12.0.0.0 10 Ajaw 8 Yaxkin	1 July 672	672	**ya-ti-ji** Paddlers
Copan	Altar H	9.13.0.0.0 8 Ajaw 8 Wo	13 March 692	March	692	9.13.0.0.0 8 Ajaw 8 Wo	18 March 692	692	**ya-ti-ji**
Tonina	Monument 134	9.13.5.0.0 1 Ajaw 3 Pop	15 February 697	February	697	9.13.5.0.0 1 Ajaw 3 Pop	20 February 697	697	**ya-ti-ji** Paddlers
Copan	Stela J west	9.13.10.0.0 7 Ajaw 3 Kumk’u	20 January 702	January	702	9.13.10.0.0 7 Ajaw 3 Kumk’u	26 January 702	702	**ya-ti-ji** Paddlers
Tonina	Monument 139	9.13.10.0.0 7 Ajaw 3 Kumk’u	20 January 702	January	702	9.13.10.0.0 7 Ajaw 3 Kumk’u	26 January 702	702	**ya-ti-ji** Paddlers
Tonina	Monument 56	9.13.15.0.0 13 Ajaw 18 Pax	25 December 706	December	706	9.13.15.0.0 13 Ajaw 18 Pax	31 December 706	706	**ya-ti-ji** Paddlers
Tonina	Monument 63	9.14.0.0.0 6 Ajaw 13 Muwan	29 November 711	November	711	?9.14.0.0.0 6 Ajaw 13 Muwan	5 December 711	711	**ya-ti-ji** Paddlers
Piedras Negras	Stela 3-right side	9.14.0.0.0 6 Ajaw 13 Muwan	29 November 711	November	711	9.14.0.0.0 6 Ajaw 13 Muwan	5 December 711	711	**ya?-ti-ji** Paddlers
Naranjo	Stela 2	9.14.0.0.0 6 Ajaw 13 Muwan	29 November 711	November	711	9.14.1.3.19 3 Kawak 2 Pop	16 February 713	713	**yatij?** Paddlers
Naranjo	Stela 23	9.14.0.0.0 6 Ajaw 13 Muwan	29 November 711	November	711	9.14.0.0.0 6 Ajaw 13 Muwan	29 November 711	711	**ya-AT-ji** Paddlers
Tonina	Monument 136	9.14.5.0.0 12 Ajaw 8 K’ank’in	2 November 716	November	716	9.14.5.0.0 12 Ajaw 8 K’ank’in	8 November 716	716	**ya-AT?/ti?-ji?** Paddlers
Guaquitepec	Stela 1	9.14.10.0.0 5 Ajaw 3 Mak	7 October 721	October	721	9.14.10.0.0 5 Ajaw 3 Mak	9 October 721	721	**ya-ti-ji**
Tonina	Monument 110	9.14.10.0.0 5 Ajaw 3 Mak	7 October 721	October	721	9.14.10.0.0 5 Ajaw 3 Mak	9 October 721	721	**ya-AT-ji** Paddlers
Tikal	Stela 24s	9.19.0.0.0 9 Ajaw 18 Mol	22 June 810	June	810	9.19.0.0.0 9 Ajaw 18 Mol	28 June 810	810	**ya-AT-ji-?**- Paddlers
Tikal	Stela 24f	9.19.0.0.0 9 Ajaw 18 Mol	22 June 810	June	810	9.19.0.0.0 9 Ajaw 18 Mol	28 June 810	810	**ya-AT?-ji** Paddlers
Ixlu	Altar 1	10.2.10.0.0 2 Ajaw 13 Chen	20 June 879	June	879	10.2.10.0.0 2 Ajaw 13 Chen	26 Jun 879	879	**ya-AT-ji?** Paddlers
Tonina	Frag. X	NA	NA	NA	NA	NA	NA	NA	**ya-ti-ji**
Tonina	Monument 42	NA	NA	NA	NA	NA	NA	NA	**ya-ti-ji** Paddlers
Tonina	Monument 138	NA	NA	NA	NA	NA	NA	NA	**ya-ti-ji**-Paddlers **u-CHOK-wa**
Copan	Papagayo	NA	NA	NA	NA	9.4.0.0.0 13 Ajaw 18 Yax	18 October 514	514	**ya-AT-ji**

**Table 2:** Additional 4 monuments with iconographical depictions of “bathing” ritual.

Site name	Monument	Clause date	Julian date	Season	Date C	Text (Dedication) date	Gregorian date	Date M	Transcription
Ixlu	Stela 1	10.1.10.0.0 4 Ajaw 13 K’ank’in	3 October	October	859	10.1.10.0.0 4 Ajaw 13 K’ank’in	9 October 859	859	depicted Paddler gods in clouds
Tikal	Stela 11	10.2.0.0.0 3 Ajaw 3 Keh	13 August 869	August	869	10.2.0.0.0 3 Ajaw 3 Keh	17 August 869	869	Depicted Paddler gods in clouds
Jimbal	Stela 1	10.2.10.0.0 2 Ajaw 13 Ch’en	20 June 879	June	879	10.2.10.0.0 2 Ajaw 13 Ch’en	26 Jun 879	879	depicted Paddler gods in clouds
Ixlu	Stela 2	10.2.10.0.0 2 Ajaw 13 Ch’en?	20 June 879	June	879	10.2.10.0.0 2 Ajaw 13 Ch’en?	26 June 879	879	depicted Paddler gods in clouds

**Table 3:** Monuments with written statements of scattering ritual

Site name	Monument number	Clause date	Julian date	Season	Date C	Text date (latest)	Gregorian date	Date M	Transcription
Yaxchilan	HS 1	8.17.2.12.5 4 Chikchan 18 Woh	10 June 379	June	379	9.16.10.0.0 1 Ajaw 3 Sip	17 March 761	761	**CHOK-? ch’a-ji?**
Quiriguá	Monument 26	9.3.0.0.0 2 Ajaw 18 Muwan	27 January 495	January	495	9.3.0.0.0 2 Ajaw 18 Muwan	30 January 495	495	**u-CHOK [ch’a]-ji**
Piedras Negras	Altar 1	9.4.0.0.0 13 Ajaw 18 Yax	14 October 514	October	514	? 9.13.0.0.0	18 March 692	692	**? CHOK-?-ya?**
Palenque	Palace XIX Throne W	9.6.7.0.0 7 Ajaw 8 K’ayab	9 February 561	February	561	9.15.5.0.0 10 Ajaw 8 Ch’en	26 July 736	736	**u-CHOK ch’a-ji**
Calakmul	Stela 33	9.7.10.0.0 6 Ajaw 13 Sak	12 October 583	October	583	9.11.5.0.0 5 Ajaw 3 Sak	18 September 657	657	**CHOK-ch’a?-ji?**
Calakmul	Stela 33	?9.8.0.0.0 5 Ajaw 3 Ch’en	20 August 593	August	593	9.11.5.0.0 5 Ajaw 3 Sak	18 September 657	657	**u-CHOK-wa ch’a-ji?**
Caracol	Stela 1	9.8.0.0.0 5 Ajaw 3 Ch’en	20 August 593	August	593	?9.8.0.0.0 5 Ajaw 3 Ch’en	24 August 593	593	**CHOK**
Naranjo	Altar 1	9.8.0.0.0 5 Ajaw 3 Ch’en	20 August 593	August	593	9.8.2.14.3 7 Ak’bal 11 Sotz’	23 May 596	596	**u-CHOK-?**
Caracol	Stela 6	9.8.10.0.0 4 Ajaw 13 Xul	29 June 603	June	603	9.8.10.0.0 Ajaw 13 Xul	4 July 603	603	**u-CHOK**
Caracol	Stela 3	9.9.10.0.0 2 Ajaw 13 Pop	16 March 623	March	623	9.10.0.0.0 1 Ajaw 8 Kayab	25 January 633	633	**CHOK-wa**
Altar de Sacrificios	Altar Stela 9	9.10.0.0.0 1 Ajaw 8 K’ayab	22 January 633	January	633	9.10.0.0.0 1 Ajaw 8 K’ayab	24 January 633	633	**u-CHOK**
La Honradez	Stela 4b	9.10.0.0.0 1 Ajaw 8 K’ayab	22 January 633	January	633	9.10.0.0.0 1 Ajaw 8 K’ayab	27 January 633	633	**u?-CHOK-wa**
Yaxchilan	Stela 3	9.10.16.10 13 7 Ben 16 Sek	5 June 649	June	649	NA	NA	NA	**CHOK-?**
Copan	Stela 3b	?9.11.0.0.0 12 Ajaw 8 Kej	9 October 652	October	652	?9.11.0.0.0 12 Ajaw 8 Kej	14 October 652	652	**CHOK?**
Copan	Stela 13	9.11.0.0.0 12 Ajaw 8 Kej	9 October 652	October	652	9.11.0.0.0 12 Ajaw 8 Kej	14 October 652	652	**u-cho-ko-wa ch’a**
Copan	Altar Stela5	9.11.0.0.0 12 Ajaw 8 Kej	9 October 652	October	652	9.11.15.0.0 4 Ajaw 13 Mol	28 July 667	667	**u-CHOK**
Copan	Altar Stela 5	9.11.0.0.0 12 Ajaw 8 Kej	9 October 652	October	652	9.12.0.0.0 10 Ajaw 8 Yaxk’in	1 July 672	672	**u-chok-??**
Palenque	Palace Tablet	9.11.0.0.0 12 Ajaw 8 Kej	9 October 652	October	652	9.14.8.14.15 9 Men 3 Yax	14 August 720	720	**u-CHOK-wa ch’a-ji**
Tonina	Monument 28	9.11.5.0.0 5 Ajaw 3 Sak	13 September 657	September	657	9.11.5.0.0 5 Ajaw 3 Sak	18 September 657	657	**u-CHOK ch’a?-ji?**
La Corona (Site Q)	Panel 3?	9.11.10.0.0 11 Ajaw 18 Ch’en	18 August 662	August	662	9.11.10.0.0 11 Ajaw 18 Ch’en	20 August 662	662	**u-BAH-ti-CHOK-ko-ji**
Rio Azul	Stela 2	9.11.10.0.0 11 Ajaw 18 Ch’en	18-Aug	August	662	9.11.10.0.0 11 Ajaw 18 Ch’en	August 662	662	**u-CHOK-wa?**
Copan	Stela 5	9.11.15.0.0 4 Ajaw 13 Mol?	23 July 667	July	667	9.11.15.0.0 4 Ajaw 13 Mol	28 July 667	667	**CHOK?**
Tonina	Monument 113	9.12.0.0.0 10 Ajaw 8 Yaxk’in	26 June 672	July	672	9.12.0.0.0 10 Ajaw 8 Yaxk’in	1 July 672	672	**u-CHOK-ch’a-ya?**
La Corona (Site Q)	Panel 1	?9.12.5.0.0 3 Ajaw 3 Xul	31 May 677	May	677	9.12.5.7.4 4 K’an 7 Mak	27 October 677	677	**u- CHOK**
Copan	Stela 6	9.12.10.0.0 9 Ajaw 18 Sotz’	5 May 682	May	682	9.12.10.0.0 9 Ajaw 18 Sotz’	10 May 682	682	**u-CHOK ch’a-ji?**
Tonina	Monument 8, side 3	9.12.10.0.0 9 Ajaw 18 Sotz’	5 May 682	May	682	9.12.10.0.0 9 Ajaw 18 Sotz’	7 May 682	682	**u-CHOK-wa**
Tonina	Monument 8, side 4	? 9.12.10.0.0 9 Ajaw 18 Sotz’	5 May 682	May	682	9.12.10.0.0 9 Ajaw 18 Sotz’	7 May 682	682	**u-CHOK- ?**
Tonina	Monument 111	9.12.16.3.12 5 Eb 20 Xul	14 June 688	June	688	9.13.0.0.0 8 Ajaw 8 Wo	18 March 692	692	**u-u-CHOK-wa ch’a-ji**
Aguateca	Stela 5	9.13.0.0.0 8 Ajaw 8 Woh	13 March 692	March	692	?9.13.0.0.0 8 Ajaw 8 Woh	18 March 692	692	**u-CHOK ch’a-ji**
Copan	Stela J West	9.13.10.0.0 7 Ajaw 3 Kumk’u	20 January 702	January	702	9.13.10.0.0 7 Ajaw 3 Kumk’u	26 January 702	702	**CHOK ch’a-ji**
Dos Pilas	Stela 1	9.13.15.0.0 13 Ajaw 18 Pax	25 December 706	December	706	9.13.15.0.0 13 Ajaw 18 Pax	31 December 706	706	**u-CHOK-wa ch’a-ji**
La Corona (Site Q)	HS 2 Block XI (El Peru, panel 8)	9.13.18.16.4 13 K’an 2 K’ank’in	29 October 710	October	710	?9.14.3.5.15? 13 K’an 2 K’ank’in	31 October 710	710	**CHOK-ka-ja**
Naranjo	Stela 23	9.13.18.9.15 1 Men 13 Yaxk’in	22 June 710	June	710	9.13.19.6.3 3 Ak’bal 16 Sip	12 April 711	711	**CHOK ti-PET-ni**
Dos Pilas	Stela 8	9.14.0.0.0 6 Ajaw 13 Muwan	29 November 711	November	711	9.12.0.10.11 13 Chuwen 19 K’ayab	25 January 673	673	**u-cho-ko-wa ch’a-ji**
Naranjo	Stela 30 back	9.14.3.0.0 7 Ajaw 18 K’ank’in	13 November 714	November	714	9.14.3.0.0 7 Ajaw 18 K’ank’in	19 November 714	714	**u-CHOK-ja?**
Dos Pilas	Stela 11	9.14.5.0.0 12 Ajaw 8 K’ankin	2 November 716	November	716	9.14.5.0.0 12 Ajaw 8 K’ankin	4 November 716	716	**u-CHOK-wa-ch’a-ji**
Dos Pilas	Stela 15	9.14.10.0.0 5 Ajaw 3 Mak	7 October 721	October	721	9.14.10.0.0 5 Ajaw 3 Mak	9 October 721	721	**u-CHOK-wa ch’a-ji**
Tonina	Monument 110	9.14.10.0.0 5 Ajaw 3 Mak	7 October 721	October	721	9.14.10.0.0 5 Ajaw 3 Mak	9 October 721	721	**u-CHOK-wa ch’a-ji?**
Tonina	Monument 7	9.14.17.9.0 1 Ajaw 3 Wo	27 February 729	February	729	9.14.17.9.0 1 Ajaw 3 Wo	5 March 729	729	**u-CHOK [ch’a]-ji**
Aguateca	Stela 3	9.15.0.0.0 4 Ajaw 13 Yax	16 August 731	August	731	9.15.0.0.0. 4 Ajaw 13 Yax	22 August 731	731	**u-CHOK [ch’a]-ji**
Aroyo de Piedra	Stela 2a	9.15.0.0.0 4 Ajaw 13 Yax	16 August 731	August	731	9.15.0.0.0 4 Ajaw 13 Yax	22 August 731	731	**u-CHOK-wa ch’a-[ji]**
Oxpemul	Stela 12	9.15.0.0.0 4 Ajaw 13 Yax	16 August 731	August	731	9.15.0.0.0 4 Ajaw 13 Yax	22 August 731	731	**u-CHOK-wa?**
Tikal	Stela 21	9.15.3.6.8 3 Lamat 6 Pax	6 December 734	December	734	9.15.5.0.0 10 Ajaw 8 Ch’en	26 July 736	736	**CHOK[i]-wa-ch’a-ji**
Tonina	Monument 164	9.15.3.15.5 11 Chikchan 18 Xul	1 June 735	June	735	9.15.5.0.0 10 Ajaw 8 Ch’en	26 July 736	736	**u-CHOK-ji**
Aguateca	Stela 2	9.15.5.0.0 10 Ajaw 8 Ch’en	20 July 736	July	736	9.15.5.0.0 10 Ajaw 8 Ch’en	26 July 736	736	**u-CHOK ch’a-ji**
Palenque	Palace XIX Throne W	9.15.5.0.0 10 Ajaw 8 Ch’en	20 July 736	July	736	9.15.5.0.0 10 Ajaw 8 Ch’en	26 July 736	736	**u-CHOK**
Aguateca	Stela 1	9.15.10.0.0 3 Ajaw 3 Mol	24 June 741	June	741	9.15.10.0.0 3 Ajaw 3 Mol	30 June 741	741	**u-CHOK ch’a-ji**
Aguateca	Stela 1	9.15.9.9.0 5 Ajaw 8 K’ayab	26 December 740	December	741	9.15.10.0.0 3 Ajaw 3 Mol	30 June 741	741	**u-CHOK-wa ch’a-ji**
Dos Pilas	Bench 01	9.15.9.9.0 5 Ajaw 8 K’ayab	26 December 740	December	741	9.15.10.17.15 7 Men 13 Yaxk’in	20 June 742	742	**CHOK-wa ch’a-ji**
Moral	Altar 2	9.15.10.0.0 3 Ajaw 3 Mol	24 June 741	June	741	?9.15.8.14.9 1 Muluk 17 Sotz’	?25 April 740	740	**u-CHOK ?**
Nim Li Punit	Stela 1	9.15.10.0.0 3 Ajaw 3 Mol	24 June 741	June	741	9.15.10.0.0 3 Ajaw 3 Mol	30 June 741	741	**u-CHOK ch’a-ji**
Dos Pilas	Stela 4	9.15.11.0.0 12 Ajaw 18 Yaxk’in	19 June 742	June	742	9.15.11.0.0 12 Ajaw 18 Yaxk’in	23-Jun	742	**u-CHOK-wa ch’a-ji**
Piedras Negras	Stela 40	9.15.14.9.13 11 Ben 16 Pax	13 December 745	December	745	9. 15.15.0.0 9 Ajaw 18 Xul	4 June 746	746	**CHOK [ch’a-ji]**
Piedras Negras	Stela 40	9.15.15.0.0 9 Ajaw 18 Xul	29 May 746	May	746	9. 15.15.0.0 9 Ajaw 18 Xul	4 June 746	746	**CHOK [ch’a-ji]**
Quiriguá	Stela S	9.15.15.0.0 9 Ajaw 18 Xul	29 May 746	May	746	9.15.15.0.0 9 Ajaw 18 Xul	4 June 746	746	**u-CHOK ?**
Seibal	HS 1	9.15.14.17.18 7 Etz’nab 16 Xul	29 May 746	May	746	9.16.0.0.0 2 Ajaw 13 Sek	5 May 751	751	**u-CHOK-wa ch’a-ji**
Seibal	HS 1	9.15.15.0.0 9 Ajaw 18 Xul	29 May 746	May	746	9.16.0.0.0 2 Ajaw 13 Sek	5 May 751	751	**u-CHOK-wa-ch’a-ji**
Calakmul	Stela 62	9.16.0.0.0 2 Ajaw 13 Sek	3 May 751	May	751	9.16.0.0.0 2 Ajaw 13 Sek	9 May 751	751	**CHOK?**
Quiriguá	Stela H East	9.16.0.0.0 2 Ajaw 13 Sek	3 May 751	May	751	9.16.0.0.0 2 Ajaw 13 Sek	5 May 751	751	**u-CHOK? ch’a-ji**
Quiriguá	Stela J East	9.16.5.0.0 8 Ajaw 8 Sotz’	6 April 756	April	756	9.16.5.0.0 8 Ajaw 8 Sotz’	8 April 756	756	**u-CHOK-ji**
Quiriguá	Stela F East	9.16.10.0.0 1 Ajaw 3 Sip	11 March 761	March	761	9.16.10.0.0 1 Ajaw 3 Sip	13 March 761	761	**cho-ka-ja-ch’a-ji**
Sacul	Stela 1	9.16.10.0.0 1 Ajaw 3 Sip	11 March 761	March	761	9. 16.10.0.0 1 Ajaw 3 Sip	17 March 761	761	**u-CHOK ch’a-[ji]**
Yaxchilan	Stela 1	9.16.10.0.0 1 Ajaw 3 Sip	11 March 761	March	761	9.16.10.0.0 1 Ajaw 3 Sip	17 March 761	761	**u-CHOK**
La Pasadita	Lintel 2	9.16.15.0.0 7 Ajaw 18 Pop	13 February 766	February	766	9.16.15.0.0 7 Ajaw 18 Pop	19 February 766	766	**u-CHOK-wa**
Quiriguá	Stela D East	9.16.15.0.0 7 Ajaw 18 Pop	13 February 766	February	766	9.16.15.0.0 7 Ajaw 18 Pop	19 February 766	766	**u-CHOK-wa ch’a-ji**
Quiriguá	Stela D West	9.16.15.0.0 7 Ajaw 18 Pop	13 February 766	February	766	9.16.15.0.0 7 Ajaw 18 Pop	19 February 766	766	**u-cho-ko-wa ch’a-ji**
Tikal	Stela 22f	9.16.17.16.4 11 K’an 12 K’ayab	23 December 768	December	768	9.17.0.0.0 13 Ajaw 18 Kumk’u	24 January 771	771	**CHOK[i] ch’a-ji**
Oxpemul	Stela 2	9.17.0.0.0 13 Ajaw 18 Kumk’u	18 January 771	January	771	9.17.0.0.0 13 Ajaw 18 Kumk’u	24 January 771	771	**u-CHOK-wa**
Pomoná	Panel 1	9.17.0.0.0 13 Ajaw 18 Kumku	18 January 771	January	771	9.17.0.0.0 13 Ajaw 18 Kumku	24 January 771	771	**u-CHOK-wa ch’a-ji**
Quiriguá	Stela E East	9.17.0.0.0 13 Ajaw 18 Kumk’u	18 January 771	January	771	9.17.0.0.0 13 Ajaw 18 Kumk’u	20 January 771	771	**u-CHOK-ch’a-ji?**
Quiriguá	Stela E West	9.17.0.0.0 13 Ajaw 18 Kumk’u	18 January 771	January	771	9.17.0.0.0 13 Ajaw 18 Kumk’u	20 January 771	771	**CHOK-wa ch’a-ji**
Quiriguá	Stela C West	9.17.5.0.0 6 Ajaw 13 K’ayab	23 December 775	December	775	9.17.5.0.0 6 Ajaw 13 K’ayab	25 December 775	775	**u-CHOK-wa**
Bonampak	Stela 1	9.17.10.0.0 12 Ajaw 8 Pax	26 November 780	November	780	9.17.10.0.0 12 Ajaw 8 Pax	2 December 780	780	**u-CHOK-ch’a-ji**
Ixkun	Stela 2	9.17.9.6.14 7 Hix 2 Sek	14 April 780	April	780	(IS) 9.17.9.0.13 3 Ben 6 K’ayab	21 December 779	779	**u-CHOK**
Ixtzutz	Stela 4	9.17.10.0.0 12 Ajaw 8 Pax	26 November 780	November	780	9.17.10.0.0 12 Ajaw 8 Pax	2 December 780	780	**u-CHOK-ko-wa ch’a-ji**
Naranjo	Stela 33	9.17.10.0.0 12 Ajaw 8 Pax	26 November 780	November	780	9.17.10.0.0 12 Ajaw 8 Pax	28 November 780	780	**u-CHOK**
Naranjo	Stela 19	9.17.10.0.0 12 Ajaw 8 Pax	26 November 780	November	780	9.17.10.0.0 12 Ajaw 8 Pax	28 November 780	780	**u-CHOK**
Bonampak	Stela 3	9.17.15.0.0 5 Ajaw 3 Muwan	31 October 785	October	785	9.15.15.3.13 13 Ben 16 Kumk’u	18 January 786	786	**u-CHOK ch’a-ji**
Quiriguá	Altar O’ c	9.17.15.0.0 5 Ajaw 3 Muwan	31 October 785	October	785	9.18.0.0.0 11 Ajaw 18 Mak	11 October 790	790	**u-u-CHOK-wa ch’a-ji**
Tikal	Stela 19f	9.17.18.3.1 2 Imix 9 K’ayab	15 December 788	December	788	9.18.0.0.0 11 Ajaw 18 Mak	11 October 790	790	**CHOK-wa-?**
Ixkun	Stela 4	?9.17.18.7.6 9 Kimi 9 Sip	10 March 789	March	789	?9.17.18.7.6 9 Kimi 9 Sip	16 March 789	789	**u?-CHOK?**
Ixkun	Stela 1	9.18.0.0.0 11 Ajaw 18 Mak	5 October 790	October	790	(IS) 9.18.0.0.0 11 Ajaw 18 Mak	11 October 790	790	**u-CHOK**
Ixkun	Stela 1	9.18.0.0.0 11 Ajaw 18 Mak	5 October 790	October	790	(IS) 9.18.0.0.0 11 Ajaw 18 Mak	11 October 790	790	**CHOK ch’a?-?**
Ixkun	Stela 1	9.18.0.0.0 11 Ajaw 18 Mak	5 October 790	October	790	(IS) 9.18.0.0.0 11 Ajaw 18 Mak	11 October 790	790	**ti-CHOK ch’a-ji?**
Naranjo	Stela 14	9.18.0.0.0 11 Ajaw 18 Mak	5 October 790	October	790	9.18.0.0.0 11 Ajaw 18 Mak	7 October 790	790	**u-CHOK-wa ch’a-[ji?]**
Nim Li Punit	Stela 21	9.18.0.0.0 11 Ajaw 18 Mak	5 October 790	October	790	9.18.0.0.0 11 Ajaw 18 Mak	11 October 790	790	**u-CHOK-wa**
Yaxha	Stela 13	9.18.3.0.0 12 Ajaw 3 Mak	19 September 793	September	793	9.18.3.0.0 12 Ajaw 3 Mak	21 September 793	793	**u-CHOK-wa? ch’a**
Quiriguá	Altar P’	9.18.5.0.0 4 Ajaw 13 Kej	9 September 795	September	795	9.18.5.0.0 4 Ajaw 13 Kej	11 September 795	795	**cho-ko-wa ch’a-ji**
Quiriguá	Zoo P north cartouche	9.18.5.0.0 4 Ajaw 13 Kej	9 September 795	September	795	9.18.5.0.0 4 Ajaw 13 Kej	11 September 795	795	**u-CHOK-ch’a**
Quiriguá	Zoo P south	9.18.5.0.0 4 Ajaw 13 Kej	9 September 795	September	795	9.18.5.0.0 4 Ajaw 13 Kej	11 September 795	795	**u-CHOK-wa ch’a-ji**
Tonina	Monument 34	9.18.5.0.0 4 Ajaw 13 Kej	9 September 795	September	795	9.18.5.0.0 4 Ajaw 13 Kej	11 September 795	795	**u-CHOK?**
Caracol	Stela 11	9.18.10.0.0 10 Ajaw 8 Sak	13 August 800	August	800	9.18.10.0.0 10 Ajaw 8 Sak	19 August 800	800	**u-CHOK-ch’a?-ji?**
Nim Li Punit	Stela 14	9.18.10. 0.0 10 Ajaw 8 Sak	13 August 800	August	800	9.18.10.0.0 10 Ajaw 8 Sak	17 August 800	800	**u-CHOK- ?**
Quiriguá	Stela I north	9.18.10.0.0 10 Ajaw 8 Sak	13 August 800	August	800	9.18.10.0.0 10 Ajaw 8 Sak	15 August 800	800	**u-CHOK-ji**
Quiriguá	Stela K south	9.18.15.0.0 3 Ajaw 3 Yax	18 July 805	July	805	9.18.15.0.0 3 Ajaw 3 Yax	20 July 805	805	**u-CHOK-?**
Quiriguá	Structure 1B-1	9.19.0.0.0 9 Ajaw 18 Mol	22 June 810	June	810	9.19.0.0.0 9 Ajaw 18 Mol	24 June 810	810	**u-CHOK-ko-wa**
Quiriguá	Structure 1B-1	9.19.0.0.0 9 Ajaw 18 Mol	22 June 810	June	810	9.19.0.0.0 9 Ajaw 18 Mol	24 June 810	810	**u-CHOK-ko-wa**
Uaxactun	Stela 7	9.19.0.0.0 9 Ajaw 18 Mol	22 June 810	June	810	9.19.0.0.0 9 Ajaw 18 Mol	28 June 810	810	**?- ch’a**
Caracol	Altar 12	9.19.10.0.0 8 Ajaw 8 Xul	30 April 820	April	820	9.19.10.0.0 8 Ajaw 8 Xul	6 May 820	820	**u-CHOK-wa?**
Itzan	Stela 6	9.19.19.16.0 6 Ajaw 18 Pop	28 January 830	January	830	9.19.19.16.0 6 Ajaw 18 Pop	21 Oct 822	822	**u-CHOK-wa? ch’a-ja**
Tonina	Monument 104	10.0.7.9.0 3 Ajaw 3 Sak	30 July 837	July	837	10.0.7.9.0 3 Ajaw 3 Sak	1 August 837	837	**u-CHOK [ch’a]-ji**
Caracol	Stela 17	10.1.0.0.0 5 Ajaw 3 K’ayab	24 November 849	November	849	10.1.0.0.0 5 Ajaw 3 K’ayab	30 November 849	849	**u-CHOK ch’a-?**
Seibal	Stela 10	10.1.0.0.0 5 Ajaw 3 K’ayab	24 November 849	November	849	10.1.0.0.0 5 Ajaw 3 K’ayab	30 November 849	849	**u-CHOK-ko-wa**
Ucanal	Stela 4?	10.1.0.0.0 5 Ajaw 3 K’ayab	24 November 849	November	849	10.1.0.0.0 5 Ajaw 3 K’ayab	30 November 849	849	**u-CHOK-wa**
Ixlu	Stela 1	10.1.10.0.0 4 Ajaw 13 K’ank’in	3 (7) October 859	October	859	10.1.10.0.0 4 Ajaw 13 K’ank’in	9 October 859	859	**u-CHOK-?-wa**
Tikal	Stela 11	10.2.0.0.0 3 Ajaw 3 Keh	13 August 869	August	869	10.2.0.0.0 3 Ajaw 3 Keh	17 August 869	869	CHOK depicted
Ixlu	Altar 1	10.2.10.0.0 2 Ajaw 13 Ch’en	20 June 879	June	879	10.2.10.0.0 2 Ajaw 13 Ch’en	26 June 879	879	**u-CHOK-ko-wa ch’a-ji**
Jimbal	Stela 1	10.2.10.0.0 2 Ajaw 13 Ch’en	20 June 879	June	879	10.2.10.0.0 2 Ajaw 13 Ch’en	26 Jun 879	879	**u-CHOK-ko-wa ch’a-ji**
Ixlu	Stela 2	10.2.10.0.0 2 Ajaw 13 Ch’en	20 June 879	June	879	10.2.10.0.0 2 Ajaw 13 Ch’en	26 June 879	879	CHOK depicted
Jimbal	Stela 2	10.3.0.0.0 1 Ajaw 3 Yaxk’in	28 April 889	April	889	10.3.0.0.0 1 Ajaw 3 Yaxk’in	4 May 889	880	**u-CHOK**
Uaxactun	Stela 12	10.3.0.0.0 1 Ajaw 3 Yaxk’in	28 April 889	April	889	10.3.0.0.0 1 Ajaw 3 Yaxk’in	4 May 889	889	**CHOK-?-ja**
Tonina	Monument 158	10.3.15.0.0 6 Ajaw 8 Sip	9 February 904	February	904	10.3.17.9.0 9 Ajaw 18 Sak	30 July 906	906	**u-CHOK-wa**
Copan	?Img0075	NA	NA	NA	NA	NA	NA	NA	**u-CHOK-wa ch’a-ji**
El Palmar	Stela 18	? 10 Ajaw 8 Sak	NA	NA	NA	NA	NA	NA	**u-CHOK-ch’a**
Coba	Stela 1	NA	NA	NA	NA	9.12.10.5.12 4 Eb 10 Yax	25 August 682	682	**? U-CHOK-? ch’a-ji?**
Jimbal	Stela 1	? Teotihuacan date?	NA	NA	NA	NA	NA	NA	**u-CHOK-ko?-ja**
Nim Li Punit	Stela 4	NA	NA	NA	NA	NA	NA	NA	**?-CHOK?**
Tortuguero	pSARC00	NA	NA	NA	NA	NA	NA	NA	**u-CHOK-?**
El Peru	Stela 39 sides	NA	NA	NA	NA	NA	NA	NA	**u-CHOK-wa?**
Jimbal	Stela 1	NA	NA	NA	NA	10.2.10.0.0 2 Ajaw 13 Ch’en	26 Jun 879	879	**u-CHOK ch’a-ji**
Lubaantun	Marker 2	NA	NA	NA	NA	9.18.0.0.0 11 Ajaw 18 Mak	11 October 790	790	**u-CHOK-wa**
Quiriguá	Stela A	millions of years	NA	NA	NA	9.17.5.0.0 6 Ajaw 13 K’ayab	29 December 775	775	**u-CHOK-ch’a**
Tonina	Monument 138	NA	NA	NA	NA	NA	NA	NA	**u-CHOK-wa**
Tonina	Monument 137	?6 Imix 9 Muwan - much earlier or much later?	Very early date	NA	NA	9.15.5.0.0 10 Ajaw 8 Ch’en	26 July 736	736	**u-CHOK-wa**
Tonina	Monument 99	NA	NA	NA	NA	NA	NA	NA	**CHOK-?**

## The seasonality of *chok* and *at* statements

Given that *chok* and *at* rituals appear to be related to agricultural practices, we examined the frequency of such statements over different months of the year (Fig. 6). Direct comparison with modern monthly rainfall totals does not demonstrate a significant association; however, the relationship between seasonal variability and occurrence of *at* statements (p=0.107) deserves further examination (Fig. 6a-b). The wettest periods are typically between May-June and September-October, with a short drier spell in August (known as the ‘meagre season’ in Belize or *canícula* elsewhere in Maya area) and a true dry season from February to April (Hastenrath 1967; Magaña *et al*. 1999). The high points of precipitation roughly correspond with high occurrences of *chok* and especially *at* statements: more precisely, there is a significantly higher frequency of *at* statements in June, October, and November (*X*^*2*^, p=0.016) and likewise significantly higher *chok* occurrences in June and October (p=0.041), even if the overall seasonality of *chok* statements seems less pronounced than for *at* (albeit in part due to differing sample sizes). A closer connection still is with present-day planting seasons in the Maya area. The first and primary planting occurs in May, at the end of the dry and beginning of the rainy season, and the second planting in October-November, especially prevalent in the humid central lowlands (i.e., Peten, Belize, Chiapas, and Tabasco) and often involves fast-ripening varieties of maize (Brewbaker 1979: 107; Nations and Nigh 1980: 10-13; Downey and Jobbov*á* 2011: 179).

Assuming there has been no dramatic change in rainfall seasonality since the Classic Maya period, it follows that local populations would rely on these months to bring the rain, especially in May before the main planting season. This is substantiated by modern practices among traditional Maya communities, where they often plant a week or two before the onslaught of the expected rains. Our suggestion is therefore that the high number of occurrences of *at* statements in June reflects situations where planting had occurred, but the expected rains had not yet arrived, and as a result rituals petitioning for rain were performed. A modern example of this behaviour was observed in Crique Sarco village in southern Belize in 2011, where locals had already planted by the end of May, but the expected rains were delayed. People were concerned and said that they would wait another few weeks, but if the rains still did not come, they would have to perform rainmaking ceremonies. Another example is a festival performed by contemporary Yucatec Maya, known as *Pa’puul*, or ‘breaking pots,’ which serves as a petition for rain in Yucatan, Mexico, and is performed on the 24^th^ of June. The festival involves frogs associated with water, with the breaking of pots producing a sound thought to evoke the clap of thunder and rain (Smithsonian National Museum of the American Indian 2012). Many aspects of the *Pa’puul* festival most likely originate in ancient Maya tradition, not least since particular period ending ceremonies involved the discard of pottery and kitchen utensils at the turn of the calendrical phase (Tozzer 1941: 151-152; see also Pendergast 1971: 9). It is clear that the occurrence of *at* statements is highly seasonal, supporting the hypothesis that these rituals were somehow involved in, or represent an early form of rain-beckoning rituals. There is less observable seasonality for the *chok* rituals, and while this may partly reflect the latter’s wider geographical and temporal distribution, it is probably more due to the fact that *chok* or ‘scattering’ rituals were more general purpose ceremonies possibly associated with annual sowing and fertility, and initially less closely related to rainfall. We can further assess the seasonality of *chok* and *at* statements by comparing them with other kinds of events mentioned in Maya texts, such as royal accession, warfare, or indeed the overall background sample constituted by all known and dated Maya texts. Maya texts in general exhibit a more random distribution across the year (Fig. 6f), whereas texts relating warfare events show a slightly greater but statistically insignificant prevalence during the dry season (Fig. 6e), which is similarly the case for accession events (Martin 2014: chart 3). Martin has suggested that the Classic Maya planned public ceremonies at times when it would be easiest to travel and when it was least likely that the ceremony would be spoiled by rain, and similarly that warfare would be more likely to take place during the dry season than the wet season when people are occupied by planting (see also Schele and Freidel 1990: 62; Martin 2014: 100, 170-174). In summary, *chok* and *at* statements exhibit higher seasonality than other kinds of texts and stronger congruence with the start of the rains and planting seasons.

## Palaeoclimate and *chok* and *at* statements

**Fig. 7:**
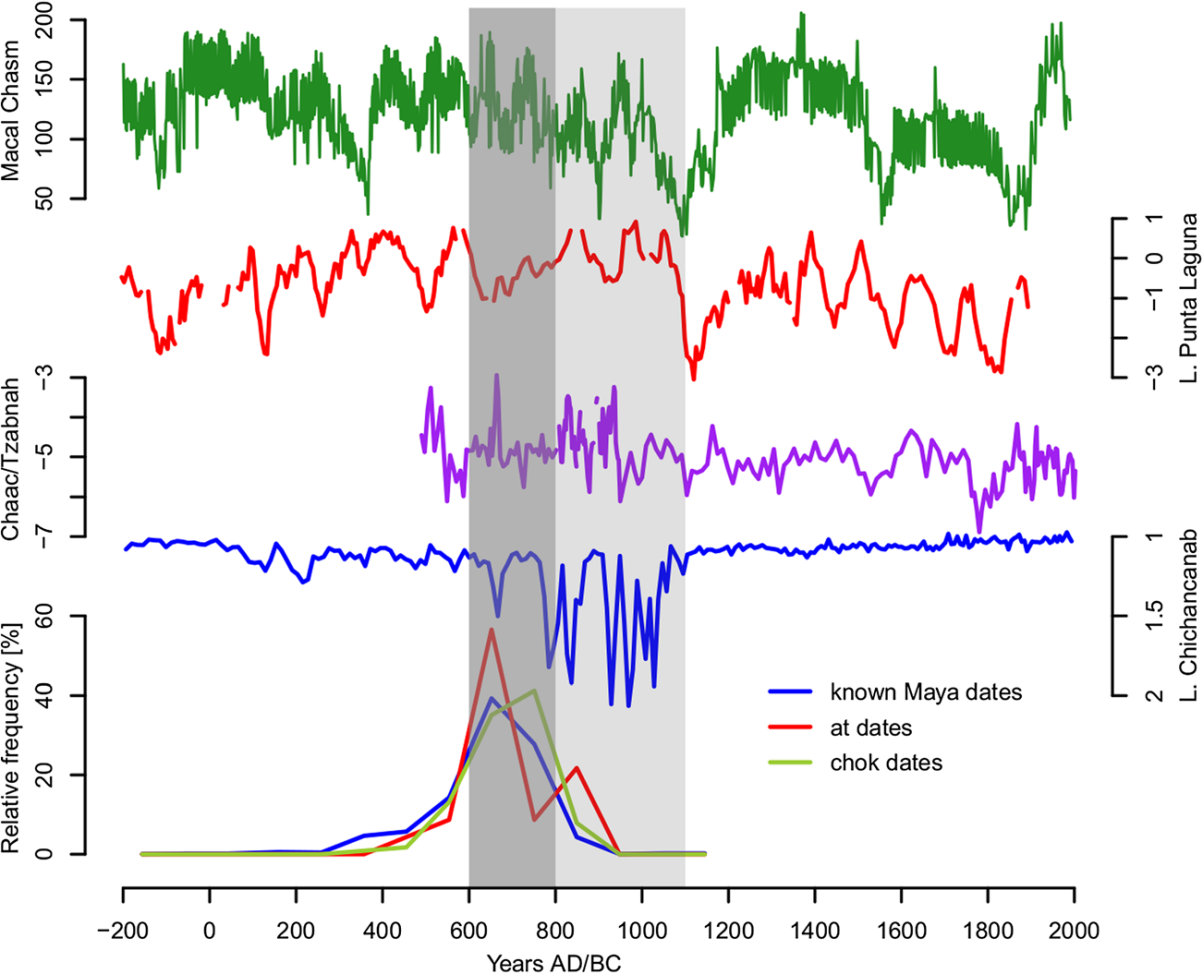
Comparison between palaeoclimatic records (Macal Chasm, Akers *et al*. 2016; Lake Punta Laguna, Curtis *et al*. 1996; Chaak/Tzabnah, Medina-Elizalde *et al*. 2010; Lake Chichancanab, Hodell *et al*. 2005) and incidences of *chok* and *at* statements. The dark gray band indicates the Late Classic wetter period and the lighter grey band indicates Terminal Classic with diminished precipitation indicated by most palaeoclimatic records. The temporal distribution of *chok* and *at* dates was compared to the temporal distribution of all known Maya dates known from the glyphic corpus to determine whether the correspondence between the appearance of the respective statements and periods with diminished precipitation is significant

**Fig. 8:**
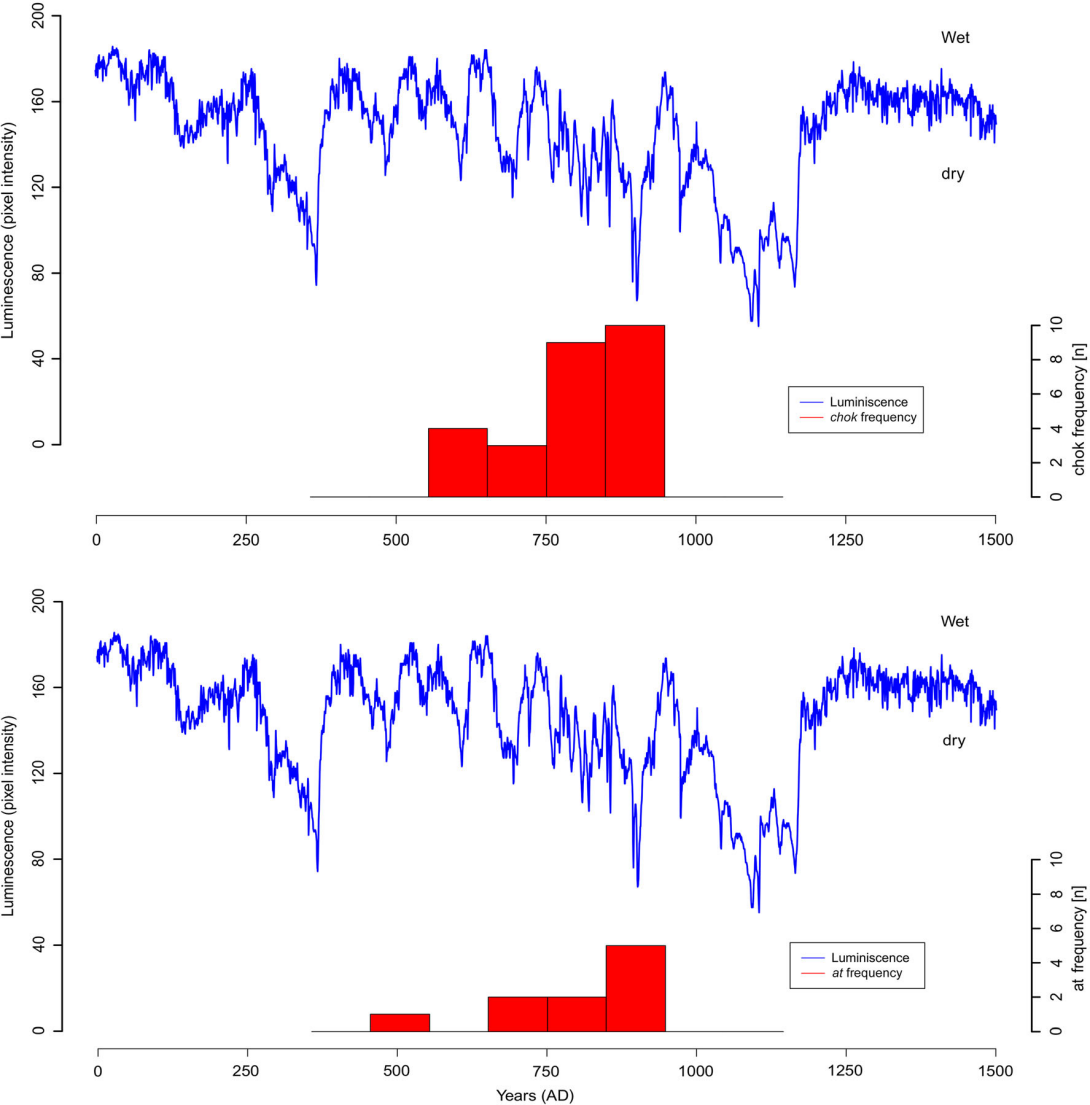
Comparison between the Macal Chasm speleothem data (Akers *et al*. 2016) and temporal distribution of *chok* and *at* statements (note that monuments with iconographic scenes are included)

Our second hypothesis is that these rituals may have originated in response to particular episodes of drought and as such could serve as markers of diminished precipitation. To address this, we compare the distribution through time of *chok* and *at* statements against palaeoclimatic evidence for periods of greater or lesser rainfall (Fig. 7). However, since there is considerable geographical variability in climatological records, the central Peten—roughly a geographic median of the Maya world—was chosen as a case study area. This region provides palaeoclimatic data from the Macal Chasm speleothem (Akers *et al*. 2016), the sites located in the area mention both *chok* and *at* rituals, and in fact produced the majority of *chok* and *at* statements during the Terminal Classic period (Fig. 8). Nevertheless, our finds suggest that the marked increase in the number of the *at* statements in the period AD 652-751 closely follows an overall increase in the incidence of hieroglyphic texts in general (Fig. 7). There is, however, a small second peak in *at* statements between AD 850 and 900 by which time the number of hieroglyphic texts in general has decreased considerably. The peak in occurrence of *chok* statements appears slightly but significantly later than the overall increase in hieroglyphic texts (KS-test, p=0.05). The palaeoclimatic data from different regions of Maya lowlands match this increased incidence of *chok* statements well (Table 4).

**Table 4:** Major droughts according to palaeoclimatic data from selected localities in the Maya area, arranged according to temporal incidence.

Locality	Dry period	Reference
Punta Laguna	AD 750-850	Curtis *et al*. 1996
Laguna Yaloch	AD 750-900	Wahl *et al.* 2013
Macal Chasm	AD 750-900	Akers *et al*. 2016
Lake Coba	AD 760-770	Hodell *et al*. 2007
Lake Chichancanab	AD 770-870	Hodell *et al*. 2005
Lake Salpeten	AD 800-900	Rosenmeier *et al*. 2002
Tzabnah Cave	AD 804-938	Medina-Elizalde *et al*. 2010
Yok Balum Cave	AD 820-870	Kennett *et al*. 2012
Lake Coba	AD 830-890	Hodell *et al*. 2007
Punta Laguna	AD 910-990	Curtis *et al*. 1996

**Fig. 9:**
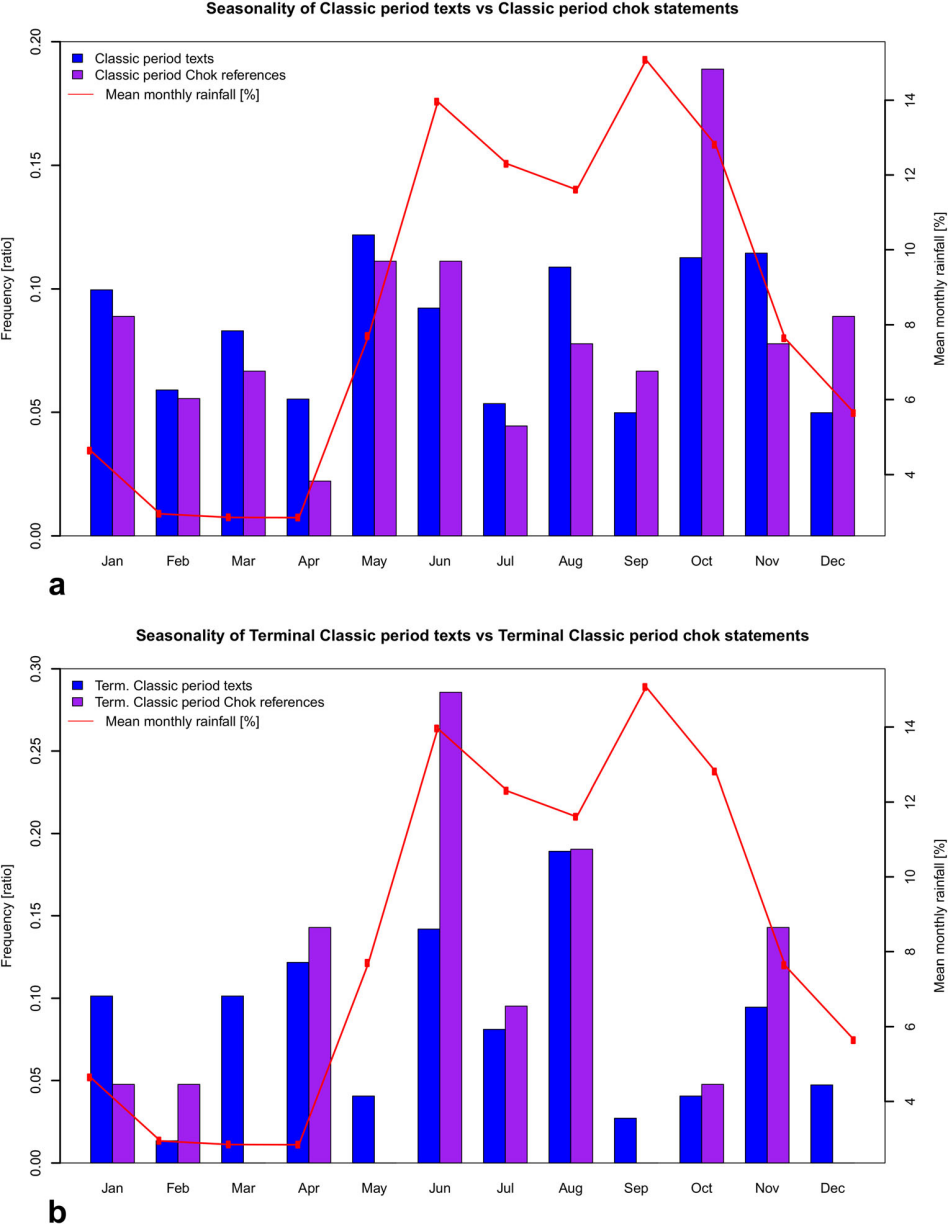
The Seasonality of *chok* statements during the Early/Late Classic and Terminal Classic periods in comparison to seasonality of dated texts during the same periods

**Fig. 10:**
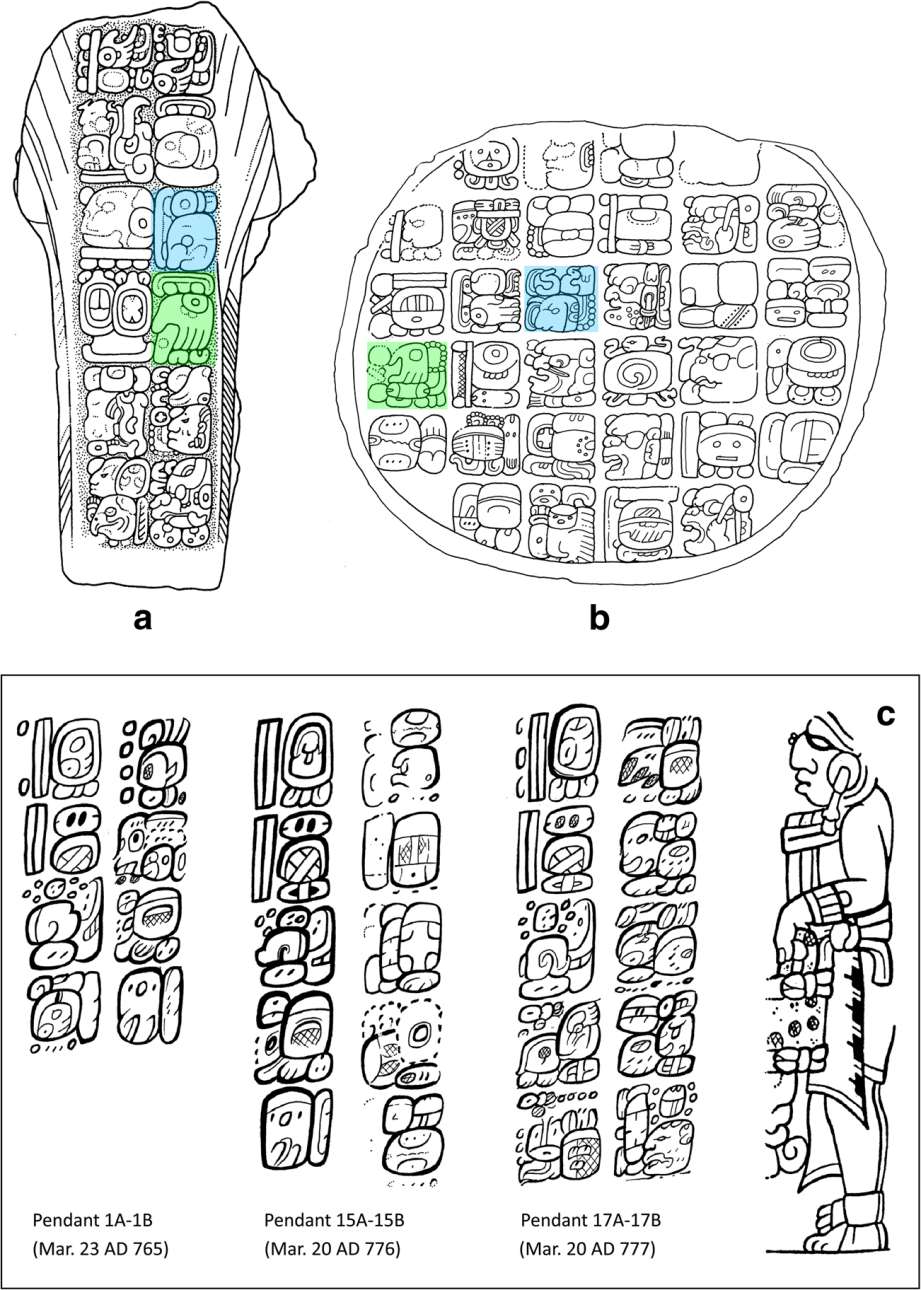
Examples of the late co-occurrence of *chok* (green) and *at* (blue) events on the same monuments. **a**) Tonina Monument 138 (drawing by Ian Graham © the President and Fellows of Harvard College, Peabody Museum of Archaeology and Ethnology, PM# 2004.15.6.16.28). **b**) Ixlu Altar 1 (drawing © Linda Schele, Los Angeles County Museum of Art). **c**) Depiction of *Ajpakal Tahn* of Comalcalco and texts commemorating some of the rituals that he performed at regular intervals, especially on the day 10 Sip in the Haab calendar. The dates in parentheses represent the proleptic Gregorian calendar (drawings by Marc Zender, after Zender 2004: Figs. 71-76)

To summarise, during the Early/Late Classic period, it seems that incidences of *chok* rituals closely follow the incidence of texts in general (Fig. 9a), suggesting that we should not interpret *chok* rituals as responses to unusual events but rather as general-purpose ceremonies performed at period-endings in the Maya calendar and/or as part of other important ceremonies, such as accessions. It is further noteworthy that *chok* rituals appear as highly hierarchical, with a focus on the ruler as the principal officiator, underscoring his key role in the social structure as a bringer of agrarian fertility. During the Terminal Classic period, however, the incidence of *chok* rituals becomes more seasonal and more closely matches that of the *at* statements (Fig. 9b), suggesting that *chok* rituals were being repurposed in this period to focus on ensuring agricultural security at risky periods of the year. Indeed, many Terminal Classic monuments suggest a pattern where *chok* and *at* rituals were performed conjointly (Fig. 10a-b, such as Jimbal Stela 1 where Jaguar Paddler is shown performing the scattering, Fig. 4a).

We also see other changes in the character of the Maya textual sources in the Terminal Classic. By AD 800 the obvious war narratives such as the texts from Naranjo, Yaxchilan, Piedras Negras, and Bonampak disappear almost altogether (Helmke *et al*. 2010: 120-121) and the period between AD 800-850 involves attempts at re-establishing and maintaining the ‘old order’ with former adversaries conducting joint rituals and visiting each other (e.g., Caracol and Ucanal, Tikal and Calakmul at Seibal) (Grube 1994: 95-97; Helmke *et al*. 2010). Between AD 850-910, there are more references to period endings and rituals (e.g., Ixlu, Jimbal, Xunantunich, Machaquila, Uaxactun, Tonina) (Schele and Grube 1995). These shifting emphases in the texts were further accompanied by changes in the composition and iconography of stelae, with a de-emphasis on the king as sole autocratic ruler, and an increased emphasis on so-called confrontation scenes, wherein power-sharing and decentralisation are apparent between multiple actors (especially pairs, Chase 1983: 105 -110) and the appearance of emblem glyphs and other royal titles at formerly secondary centres (Martin and Grube 2000: 98-99; Rice and Rice 2004: 133-134; Valdés and Fahsen 2004; Zrałka 2008: 200; Helmke *et al*. 2010: 109-110; see also Murphy et al. (2016) who argue that the increased frequency of agriculture related rituals and diminished accession and ruler-focused rituals are related to change in political organisation between Maya Classic and Postclassic period). In addition, there are also changes in settlement patterns and in many cases a decrease in overall population (e.g., for the Belize River Valley see Willey *et al*. 1965; Ford 1990; LeCount and Yaeger 2010; Hoggarth 2012; for the Peten Lakes see Rice and Rice 1980, 2004; Rice 1986).

## Spatial patterning of *chok* and *at* statements

**Fig. 11:**
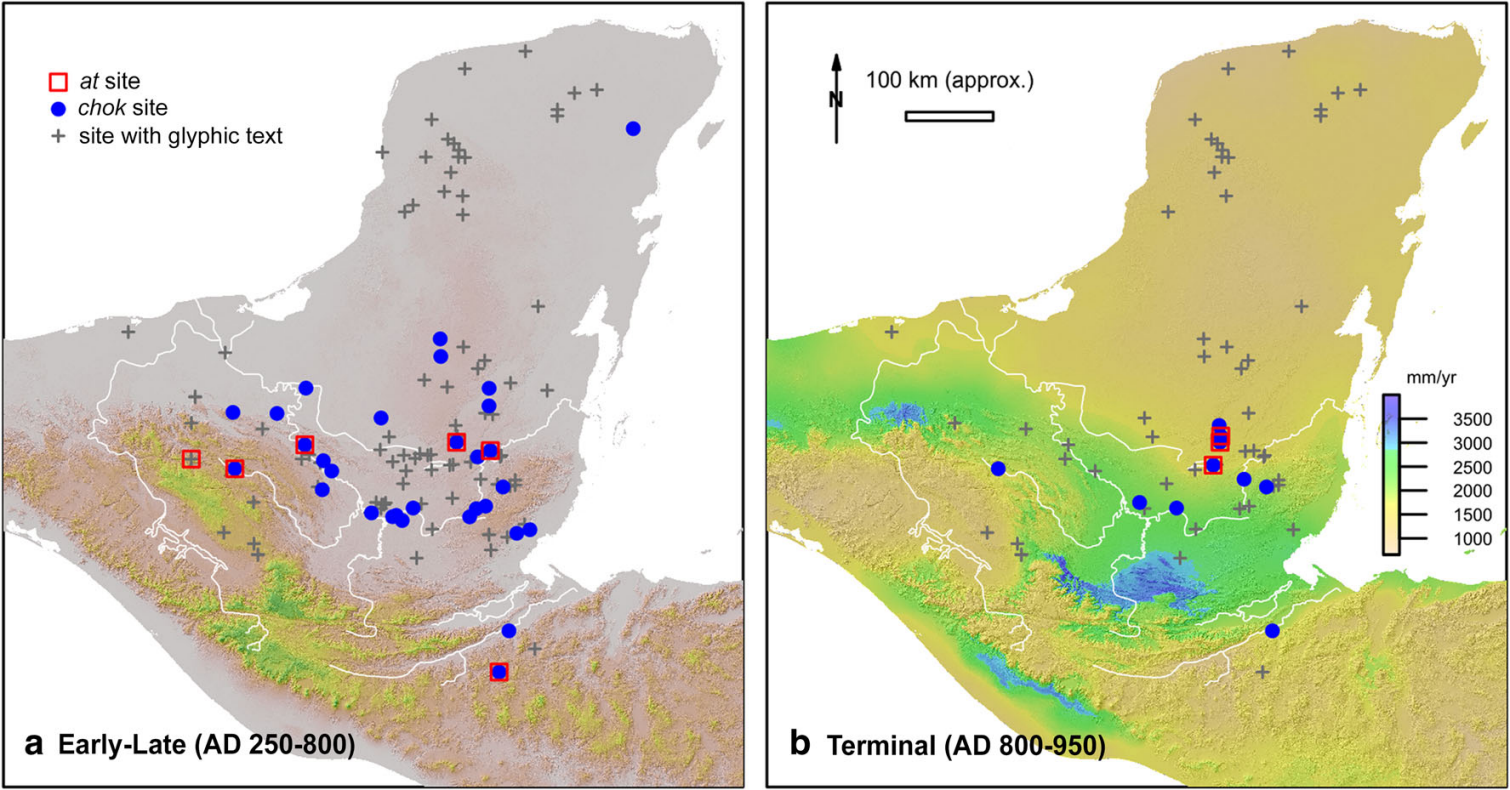
**a**) Distribution of *chok* and *at* statements of the Early and Late Classic against the distribution of all sites with glyphic texts. **b**) Distribution of Terminal Classic period *chok* and *at* statements against modern annual rainfall patterns (Elevation model from NASA SRTM; rainfall distribution from www.worldclim.org)

A further important feature of a number of *chok* and *at* statements is that they do not occur evenly across the whole Maya world with certain sites producing such statements well in excess of the number of hieroglyphic texts at these sites in general (*X*^*2*^, p=5.9e-8, p=2.8e-24). If we further compare the distribution of *at* statements against the map of modern precipitation values (Fig. 11b), all sites with *at* statements are predominantly located in areas with moderate totals of annual rainfall, especially the sites in Peten (i.e., Tikal, Ixlu, Jimbal and Naranjo), but also Copan in Honduras. More precisely, they can be characterised as falling into something of a ‘goldilocks’ zone between high and low annual precipitation regimes, with variation in rainfall likely to have considerable effect both inter-annually and over longer time periods. Furthermore, sites with *at* rituals are located in areas without easy access to groundwater and in the case of Tikal and Jimbal also without surface water sources. It is therefore possible that ‘bathing’ rituals were perceived of as more important in this region with unpredictable but highly consequential environmental stress than either in the rain-poor, but groundwater rich northwest Yucatan, or the rain-rich southern highlands. If we consider only *chok* (‘scattering’) statements, the Early/Late Classic period distribution (Fig. 11a) is much more regionally variable, but more closely matches the clustering of the *at* statements in a core geographical region during the Terminal Classic (Fig. 11b).

Beyond this general geographic patterning, it is worth emphasising that the incidence of these rituals was likely further conditioned by individual actors at specific places on specific occasions. For example, Tonina contributes 9 of the 23 known *at* expressions, and the six datable examples from this site occur within a span of only 24 years. The Tonina examples are also performed at regular intervals, corresponding to the celebration of so-called hotun intervals of about five years (i.e., AD 697, 702*, 706*, 711, 716, 721),^3^ suggesting that a single ritual specialist might have been responsible for the entire set in much the same way as Comalcalco texts commemorate the rites performed by a single priest named *Ajpakal Tahn* over a period of 12 years (Fig. 10c, Zender 2004: 250).

## Conclusions

There is a close correspondence between the occurrence of *at* and *chok* statements and the onset of rainy seasons. This strongly supports our hypothesis that these rituals were closely related to the agrarian cycle, symbolizing the act of sowing, and the invocation of rain-bearing clouds. The frequency of *at* (‘bathing’) statements through time matches the frequency of surviving Maya texts overall, indicating that Classic period examples of ‘bathing’ rituals were an already well-established tradition that perhaps was even performed annually (in a wider agricultural context) at the end of the dry season and before the second planting. There is a small second peak in *at* statements during the Terminal Classic, corresponding with a statistically-significant increase of *chok* rituals. This also coincides with other changes in the Terminal Classic, such as shifts in narrative form and content, as well as changes in settlement patterns, all of which can now be linked in various ways to palaeoclimatic records suggesting a period of more frequent droughts. More precisely, the increased seasonality of ‘scattering’ statements and their appearance alongside ‘bathing’ rituals in Terminal Classic texts suggest that the focus of these rituals became more narrowly-focused on food security and rain-making, particularly for sites lying in the Maya heartland where diminished or delayed precipitation would have had considerable adverse consequences.

## Footnotes

1 The text is headed by a calendar round that is anchored to the 17^th^ k’atun, or the 9.17.0.0.0 Period Ending, corresponding to AD 771. The mention of the ‘thirteenth year’ can be interpreted as either 9.17.13.0.0 or AD 783, as the authors prefer, or alternatively within the 17^th^ k’atun as 9.16.13.0.0 or AD 763 (Zender 2004: 257; see also Guenter 2014: 286).

2 Approval for human subjects research was received from the University of Arizona Human Subjects Protection Program (No. 09-0418-02) and the Institute for Social and Cultural Research (Permit No. ISRC/H/2/5).

3 The dates marked with asterisks conform to this five-year pattern since these events were performed on 20^th^ of January 702 and on the 25^th^ of December 706.
